# Estrogen deficiency reduces maximal running capacity and affects serotonin levels differently in the hippocampus and nucleus accumbens in response to acute exercise

**DOI:** 10.3389/fnins.2024.1399229

**Published:** 2024-06-25

**Authors:** Earric Lee, Tuuli A. Nissinen, Laura Ylä-Outinen, Aaro Jalkanen, Jari E. Karppinen, Victoria Jeanne Vieira-Potter, Arto Lipponen, Sira Karvinen

**Affiliations:** ^1^Faculty of Sport and Health Sciences, University of Jyväskylä, Jyväskylä, Finland; ^2^Montreal Heart Institute, Montréal, QC, Canada; ^3^School of Kinesiology and Physical Activity Sciences, University of Montréal, Montréal, QC, Canada; ^4^Faculty of Health Sciences, School of Pharmacy, University of Eastern Finland, Kuopio, Finland; ^5^Obesity Research Unit, Research Program for Clinical and Molecular Metabolism, Faculty of Medicine, University of Helsinki, Helsinki, Finland; ^6^Division of Foods, Nutrition and Exercise Sciences, Department of Nutrition and Exercise Physiology, University of Missouri, Columbia, MO, United States; ^7^Department of Psychology, Faculty of Education and Psychology, University of Jyväskylä, Jyväskylä, Finland; ^8^Gerontology Research Center and Faculty of Sport and Health Sciences, University of Jyväskylä, Jyväskylä, Finland

**Keywords:** ovariectomy, menopause, neurochemical marker, energy expenditure, skeletal muscle, adipose tissue

## Abstract

**Introduction:**

Estrogen deficiency is associated with unfavorable changes in body composition and metabolic health. While physical activity ameliorates several of the negative effects, loss of ovarian function is associated with decreased physical activity levels. It has been proposed that the changes in brain neurochemical levels and /or impaired skeletal muscle function may underlie this phenomenon.

**Methods:**

We studied the effect of estrogen deficiency induced via ovariectomy (OVX) in female Wistar rats (*n* = 64). Rats underwent either sham or OVX surgery and were allocated thereafter into four groups matched for body mass and maximal running capacity: sham/control, sham/max, OVX/control, and OVX/max, of which the max groups had maximal running test before euthanasia to induce acute response to exercise. Metabolism, spontaneous activity, and maximal running capacity were measured before (PRE) and after (POST) the surgeries. Three months following the surgery, rats were euthanized, and blood and tissue samples harvested. Proteins were analyzed from gastrocnemius muscle and retroperitoneal adipose tissue via Western blot. Brain neurochemical markers were measured from nucleus accumbens (NA) and hippocampus (HC) using ultra-high performance liquid chromatography.

**Results:**

OVX had lower basal energy expenditure and higher body mass and retroperitoneal adipose tissue mass compared with sham group (*p* ≤ 0.005). OVX reduced maximal running capacity by 17% (*p* = 0.005) with no changes in muscle mass or phosphorylated form of regulatory light chain (pRLC) in gastrocnemius muscle. OVX was associated with lower serotonin metabolite 5-hydroxyindoleacetic acid (5-HIAA) level in the NA compared with sham (*p* = 0.007). In response to acute exercise, OVX was associated with low serotonin level in the HC and high level in the NA (*p* ≤ 0.024).

**Discussion:**

Our results highlight that OVX reduces maximal running capacity and affects the response of brain neurochemical levels to acute exercise in a brain region-specific manner. These results may offer mechanistic insight into why OVX reduces willingness to exercise.

## 1 Introduction

Withdrawal of ovarian hormones by ovariectomy (OVX) in rodents is commonly used to mimic the effects of menopause in the female body. Loss of ovarian function in women and female rats is associated with increased body weight and adipose tissue mass, as well as a decline in metabolic health (Ley et al., 1992; Asarian and Geary, 2002; de Souza et al., 2019; Juppi et al., 2022). However, the mediating factors are not precisely known. Due to increasing life expectancy, women can live more than one-third of their lives in an estrogen deficient state (Peacock and Ketvertis, 2023). Consequently, it has become more important than ever to understand the mechanisms and find solutions to counteract the possible adverse effects of menopause.

We and others have shown that physical activity ameliorates several negative effects associated with menopause in women (Behall et al., 2003; Karvinen et al., 2019; Juppi et al., 2022). However, the loss of ovarian hormones has been associated with decreased physical activity levels in rodents (Chen et al., 2014; Park et al., 2016), and similar findings have been linked to menopause (Pettee Gabriel et al., 2015; Laakkonen et al., 2017). Moreover, estrogen deficiency has also been associated with changes in brain serotonin and dopamine levels (Meeusen and De Meirleir, 1995; Izumo et al., 2012) and/or impaired skeletal muscle function (Moran et al., 2006; Qaisar et al., 2013; Phung et al., 2018). While several studies have reported a decrease in spontaneous physical activity (Giles et al., 2010; Izumo et al., 2012) and voluntary wheel running (Kadi et al., 2002; Hertrampf et al., 2006; Park et al., 2016) after OVX in rodents, the loss of ovarian hormones and its effect on maximal running capacity has yet to be determined. Furthermore, whether alterations in muscle myosin activity or brain neurochemical marker levels are associated with potential changes in running capacity remains unexplored.

Loss of ovarian function has important effects on neurochemical marker production and release (Long et al., 2018). The brain dopamine system has been considered a key controller for the regulation of motivation contributing to voluntary physical activity (Owesson-White et al., 2008; Park et al., 2016). Mood and motivation are associated with brain serotonin (Meyniel et al., 2016), which is involved in numerous physiological processes including motor and cognitive activities (Jacobs and Fornal, 1997; Švob Štrac et al., 2016). Lack of serotonin has been suggested to play a role in depression and anxiety (Moncrieff et al., 2022), whilst acute bouts of exercise have been shown to increase brain serotonin system function (Chaouloff, 1997; Young, 2007). Estrogens have been shown to increase the concentration of serotonin and to modulate serotonin action by regulating the distribution and state of its receptors (Wissink et al., 2001; Rybaczyk et al., 2005). Of the brain regions, the hippocampus (HC) has a major role in learning and memory, while nucleus accumbens (NA) is considered as the neural interface between motivation and action (Jarrard, 1993; Ikemoto and Panksepp, 1999). Previous studies in rodents have reported both OVX and exercise-induced changes in the HC and NA (Heikkinen et al., 2002; Erickson et al., 2011; Park et al., 2016; Chen et al., 2019). Although an acute bout of exercise alters various neurochemical marker levels in the brain (Meeusen and De Meirleir, 1995), whether OVX modulates this response has yet to be fully investigated. In addition, few studies have compared brain regions for how ovarian hormones and exercise affect neurochemical release, and how brain region-specific changes in neurochemicals may mediate changes in exercise behavior.

We and others have previously shown that loss of ovarian hormones impairs skeletal muscle function and may contribute to the loss of muscle mass associated with menopause (Moran et al., 2006; Phung et al., 2018; Karvinen et al., 2021). More specifically, estrogen affects myosin regulatory light chain (RLC) activity (Lai et al., 2016). According to this observation, OVX leads to a situation where fewer myosin heads are available to trigger muscle contraction. We have also shown that OVX may induce muscle atrophy through microRNA signaling, possibly leading to decreased muscle mass (Karvinen et al., 2021). These changes may partly underlie the decrease in physical activity levels observed following the decline of ovarian hormones. Of the lower limb muscles, the gastrocnemius is the largest muscle contributing to locomotion, and thus extensively studied in the context of treadmill running (Tsai et al., 2012; Alexander et al., 2021). Hence, changes in pRLC level may also affect maximal running capacity because of reduced force production from the gastrocnemius muscle. Menopause is widely believed to reduce energy expenditure (Xu and López, 2018), even though recent literature challenges this topic (Pontzer et al., 2021; Karppinen et al., 2023). At cellular level, the amount and efficiency of mitochondria and uncoupling proteins (UCP) play the main role in thermogenesis, and hence, contribute to energy expenditure (Rousset et al., 2004). UCP family contains UCP1, UCP2, and UCP3, which localize to mitochondrial inner membrane and uncouple oxidative phosphorylation leading to energy dissipation as heat (Rousset et al., 2004; Riley et al., 2016). A previous study showed that estrogen deficiency is associated with lower UCP2 mRNA expression in white adipose tissue of female rats (Pedersen et al., 2001). Furthermore, it has been suggested that UCP1 is protective against metabolic dysfunction associated with loss of ovarian hormones (Clookey et al., 2018).

The purpose of the present study was to investigate whether OVX reduces maximal running capacity in female rats via affecting brain neurochemical levels and/or skeletal muscle RLC activation. In agreement with previous literature, OVX led to increased body and adipose tissue masses compared with a sham operation. OVX rats had similar energy intake and spontaneous activity levels, but lower energy expenditure compared with sham rats, suggesting that the adipose tissue mass increase resulted from decreased basal energy expenditure. OVX was also associated with low UCP3 levels in gastrocnemius muscle. In support of our hypothesis, we report that OVX led to reduced maximal running capacity, although did not affect lower limb muscle mass or the level of pRLC in the gastrocnemius muscle. Interestingly, we found that OVX was associated with low serotonin levels in the HC and high level in the NA in response to acute exercise, which may offer mechanistic insight into why OVX consistently reduces willingness to run in rodents.

## 2 Materials and methods

### 2.1 Ethical approval

The animal experiment was approved by the national Project Authorization Board (ELLA, Finland, permit number ESAVI/4209/2021). All procedures with the animals were conducted in accordance with the “Principles of Laboratory Animal Care” (NIH publication #85–23, revised in 1985) and the European Commission Directive 2010/63/EU. All efforts were made to minimize the number of animals used and their suffering.

### 2.2 Rat model

A total of 64 female Wistar (Hans) rats were purchased from Envigo (Indianapolis, United States, bred and shipped from the Netherlands). Rats arrived at the animal unit of the University of Jyväskylä at the age of 13–15 weeks (~3 months). All rats were housed 2/cage in an environment-controlled facility (12/12 h light–dark cycle, 22°C) and received water and estrogen-free rodent feed (2019X, Envigo, Indianapolis, United States) *ad libitum* upon the arrival to the animal unit. During the recovery period from the surgeries (1 week) and measurements of spontaneous activity and energy and water intake, the rats were housed individually.

### 2.3 Study design

**Figure 1:**
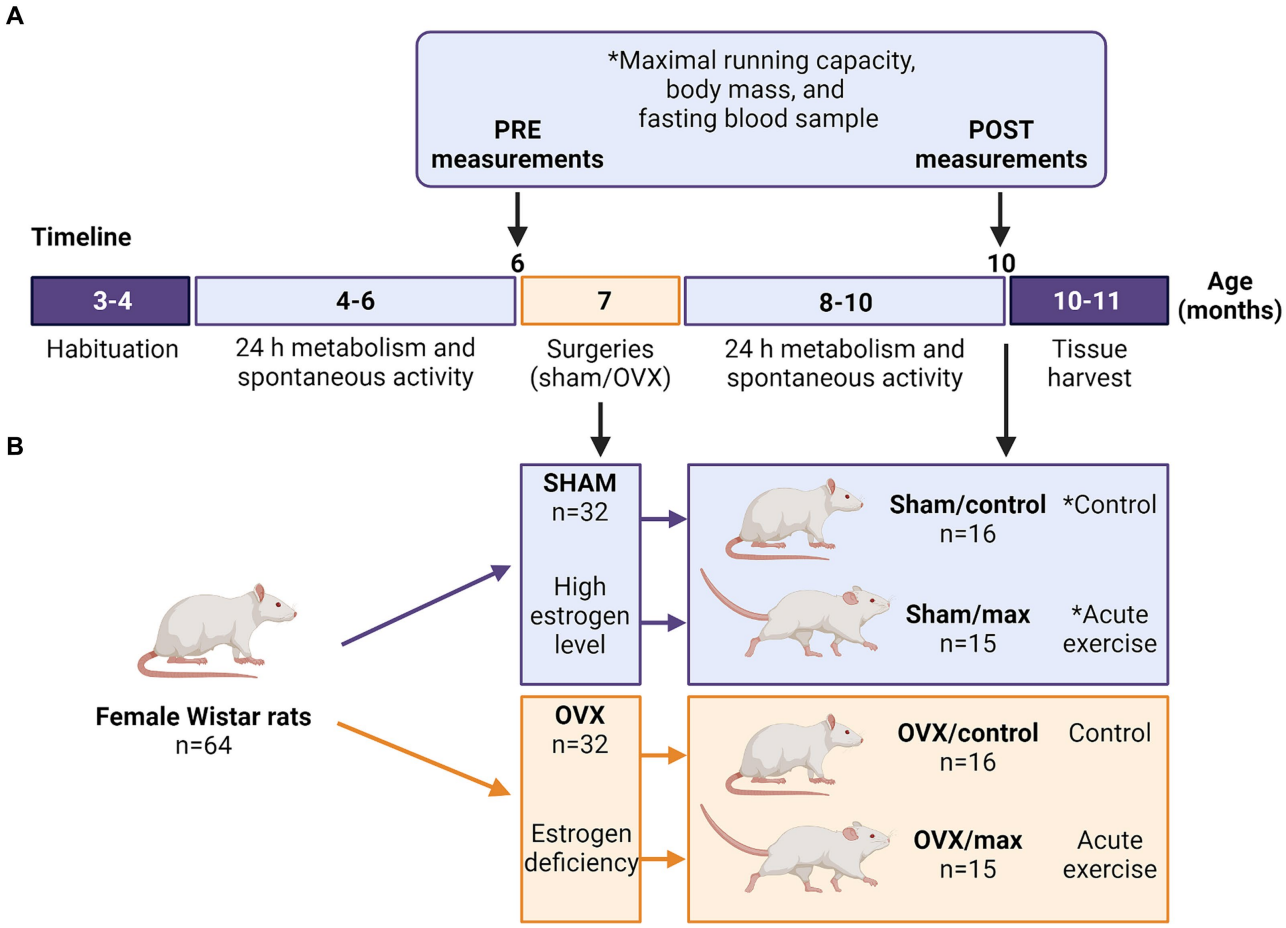
Study timeline with measurements (**A**) and study groups (**B**). Sham, ovary-intact, OVX, ovariectomy, ^*^cytology sample collected to assess the phase of estrous cycle (before PRE maximal running tests and from sham groups before euthanasia). Created with BioRender.com.

After arriving at the animal unit, the rats had 1 month of habituation to the new environment (Figure 1A). PRE measurements were performed when the rats were 4–6 months old. The measured outcomes were body mass, energy expenditure, fasting blood parameters (glucose, insulin), maximal running capacity, respiratory exchange ratio (RER) and spontaneous activity during 24 h metabolic cage stay (Figure 1A). Rats were then divided into sham and OVX groups matched for body mass and maximal running capacity (*n* = 32/group). Sham and OVX surgeries were performed when the rats were 7 months old to ensure that they were fully grown before the interventions (Yousefzadeh et al., 2020) (Figure 1A). Two animals were euthanized because of post-surgery complications (final *n* = 62). After 2 months (57 ± 4.8 days) of recovery, POST metabolism and spontaneous activity measurements were carried out prior to other follow-up measurements (Figure 1A). Thereafter, rats were further divided into control and maximal running capacity (max) groups (*n* = 15–16/group), which were matched for body mass and maximal running capacity within a group (sham or OVX) according to the POST measurements (Figure 1B). Euthanasia and tissue harvest were performed at the age of 10–11 months, on average 3 months (104 ± 4.8 days) after the surgery (Figure 1A). Rats in the sham group were euthanized at proestrus (determined by cytology sample) to ensure that they were in the highest possible systemic estrogen state.

### 2.4 Identifying the phase of the estrous cycle

The phases of the estrous cycle (proestrus, estrus, metestrus or diestrus) were identified from vaginal mucosa samples using Giemsa stain (09204, Merck, NY, United States) and according to the appearance of nucleated or anucleated epithelial cells and leucocytes (Supplementary Figure S2B). Rats in the sham group were euthanized at proestrus to ensure high systemic estrogen levels (McLean et al., 2012; Frasier et al., 2013). The phase of the estrous cycle was also determined at the time of the PRE maximal running tests.

### 2.5 Metabolism and spontaneous activity

Energy expenditure and RER were assessed with indirect calorimetry during a 24 h metabolic cage stay as described previously (Lensu et al., 2020). Briefly, the commercial metabolic cages were equipped with Promethion^®^GA3 gas analyzers (Sable Systems, Las Vegas, NV, United States) and FR8 flow generators (Sable Systems) (Lensu et al., 2020). The flow rate was set at 3,000 mL/min and the raw data were processed using ExpeData^®^ software (Sable Systems). The Weir equation was used to calculate energy expenditure (kcal/h) = 60 × (0.003941 × V̇O_2_ + 0.001106 × V̇CO_2_). The metabolic cages were also equipped with ground-reaction force recording systems to record spontaneous physical activity as described previously (Silvennoinen et al., 2014). To obtain a single value for total spontaneous activity (activity index), 1-s means were summed for the total measurement time, and the sum was divided by the body mass (kg) of the measured rat. Each rat was measured PRE and POST the sham/OVX surgery, with at least 1 month of recovery from the surgery before the follow-up measurement. The metabolism outcomes and spontaneous activity were determined for the whole 24 h measurement time as well as for the dark (12 h) and light (12 h) periods because rats are active during dark periods. Two OVX and one sham group rat exhibited a substantial increase in energy expenditure from PRE to POST compared with the other animals within their respective groups (>3 × interquartile range). We opted for a conservative approach and excluded these animals from the statistical analyses because the applied statistical methods were sensitive to extreme outliers.

### 2.6 Maximal running capacity and fasting blood measurements

Rats were tested for their maximal running capacity with a speed-ramped treadmill running test (15° slope, initial velocity of 10 m/min, increased 1 m/min every 2 min) at the age of 6 months as described previously (Koch and Britton, 2001). Rats were first habituated to running on the treadmill with three different sessions lasting for 10 min with a low velocity (<10 m/min). Maximal running test was repeated three times with at least 1 day of recovery in between. The best result of the three trials [maximal running distance (m)] was considered the maximal running capacity. Maximal running capacity was assessed during PRE and POST measurements and in addition, rats allocated into max groups (sham/max and OVX/max) performed last maximal running tests immediately before euthanasia. Since OVX rats tend to gain more adipose tissue than sham rats, the work (J) that the rats performed during the maximal running test was calculated with the following equation: Work (J) = Ascent (m) × 9.81 (N/kg) × Body mass (kg). Fasting blood glucose and insulin levels were measured to examine the effect of OVX on glucose metabolism and insulin action. Blood glucose was measured after 12 h fasting from blood harvested from the saphenous vein with a glucose analyzer (Hemocue^®^ Glucose 201). Insulin was measured with ELISA from frozen (−80°C) serum samples (Mercodia, Rat Insulin ELISA). Homeostatic Model Assessment for Insulin Resistance (HOMA-IR) was calculated with the following equation: [Glucose (mmol/l) × Insulin (μg/l)]/2.43 (Cacho et al., 2008).

### 2.7 Ovariectomy and sham surgeries

Surgeries (sham = ovary-intact or OVX = ovariectomy) were performed when the rats were 7 months old. Briefly, rats were first anesthetized with isoflurane with first induction with 5% in 450 mL/min of pressurized air followed by stable anesthesia with 2% in 450 mL/min. Then, back of the animal was shaved and disinfected with repeated swapping with iodine solution (polyvinylpyrroliodine, iodine 7.5 g/kg, Jodopax, Phrmaxim AB, Helsinborg, Sweden) and 70% ethanol. One incision was done vertically at the center of the back followed by small incision at each side on fascia to reach the ovaries. When performing OVX surgery, the ovary was ligated and removed. Thereafter the fascia and skin were closed with sutures. When performing sham surgery, each step was done similarly as in OVX surgery, except that the ovaries were not removed. Rats received subcutaneous injections of buprenorphine (0.01–0.1 mg/kg, Vetergesic vet 0,3 mg/mL, Ceva Santé Animale, France) before the surgery and every 6–12 h after the surgery for the first two days and carprofen (5 mg/kg, Rimadyl vet 50 mg/mL, Zoetis Animal Health ApS, Denmark) before the surgery and every 24 h for the first 3 days after the surgery. The possible experience of pain was assessed using the Grimace scale (Sotocinal et al., 2011). After the surgeries, the rats were housed individually for a week to ensure that the wounds healed appropriately.

### 2.8 Body mass and energy and water intake

Body mass was followed throughout the study by weighing the rats once a week. Energy and water intakes were recorded for 1 week PRE and POST surgeries when the rats were housed individually. Energy and water intake were also followed between weeks 7–14 after the surgeries when the rats were housed in pairs with both rats belonging to the same study group (sham or OVX). The energy intake was calculated from the feed energy content information provided by the manufacturer (2019X, Envigo, 3.3 kcal/g). Since the rats were housed 2/cage (with a rat from the same group), energy intake was calculated as an average of two rats.

### 2.9 Neurochemical marker measurements from brain regions with UHPCL

After the animal was euthanized, the brain was immediately removed from the skull and sliced into 1 mm thick coronal brain slices with the help of the coronal brain matrix (WPI). The coronal sections were placed on cold saline-soaked filter paper on an ice bath, and brain regions were dissected using a reusable biopsy punch, 1 mm (WPI). The collected brain samples were stored in individual tubes at −20°C until further analysis.

Seven different neurochemical markers were assayed from two brain regions, i.e., the nucleus accumbens (NA) and hippocampus (HC): serotonin, serotonin metabolite 5-hydroxyindoleacetic acid (5-HIAA), dopamine, dopamine precursor l-3,4-dihydroxyphenylalanine (L-DOPA), dopamine metabolites homovanillic acid (HVA), and 3,4-dihydroxyphenylacetic acid (DOPAC), and norepinephrine. Of the seven neurochemical markers all were detectable in the NA and four (serotonin, 5-HIAA, dopamine and norepinephrine) in the HC.

The frozen brain tissue samples were first thawed on ice and homogenized in 25 volumes (w/v) of ice-cold 0.1 M perchloric acid (Merck KGaA, Darmstadt, Germany) using an ultrasound homogenizer (Soniprep 150, MSE Scientific Instruments, Sussex, England). The homogenates were centrifuged (15,000 × g, 20 min, +4°C) and the supernatant was filtered with a 0.2 μm Acrodisc syringe filter (Pall Corporation, United States). The filtrates were further diluted with ultrapure water (NA samples 1:15 and HC samples 1:8) before analysis. The monoamine and metabolite levels were assayed using an ultra-high performance liquid chromatography (UHPLC) method with electrochemical detection (Antec Alexys Monoamine Analyzer, Antec Scientific, Hoorn, the Netherlands). The analyzer consisted of an AS 110 autosampler (set to +4°C), two LC 110S pumps (channel 1 and channel 2), an OR 110 degasser unit with pulse damper, and a DECADE II electrochemical detector equipped with two VT-03 2 mm glassy carbon working electrodes and Ag/AgCl reference electrodes. Channel 1 was used for the analysis of dopamine and serotonin and channel 2 for the analysis of norepinephrine, L-DOPA, 5-HIAA, DOPAC, and HVA. Injection volume was 10 μL. Instrument control and data acquisition were done with Clarity data system (DataApex, Prague, the Czech Republic). The chromatographic conditions for channel 1 consisted of a C18 reversed-phase column (NeuroSep 105; 50 mm × 1.0 mm, 3 μm spherical particles, Antec Scientific) and the mobile phase (50 mmol/L phosphoric acid, 0.1 mmol/L EDTA, 600 mg/L octane sulfonic acid, 12% v/v acetonitrile, pH was adjusted to 6.0 with 50% NaOH). Flow rate was 50 μL/min and pressure 54 bar. For separation of the analytes in channel 2, an ALF-115 column (150 mm × 1.0 mm, 3 μm, C18, Antec Scientific) was used. The mobile phase contained 50 mmol/L phosphoric acid, 50 mmol/L citric acid, 0.1 mmol/L EDTA, 600 mg/L octane sulfonic acid, pH 3.0, and 6% v/v acetonitrile. Flow rate was 75 μL/min and pressure 194 bar. The columns and detectors were kept at 35°C by a column oven. The applied potential was set to 0.46 V and 0.80 V for channel 1 and 2, respectively. Analyte amounts producing a detector signal with a signal-to-noise ratio > 3 was considered the limit of detection (LOD). For each analyte, the linear (r^2^ > 0.999) calibration curve was ranging from 0.5 to 50 nM, the lower limit of quantification (LLOQ) was 0.5 nM, and the quality control samples for low, intermediate, and high concentration levels were within the validated range of ±15% of nominal concentrations. Before performing the statistical analysis, the extreme outliers were first excluded (>3× interquartile range).

### 2.10 Tissue and plasma harvest

At the end of the study, rats were euthanized after a 2 h fast using carbon dioxide followed by heart puncture. Tissues (heart, liver, adipose tissue, and skeletal muscles) were weighed, snap frozen in liquid nitrogen and stored at −80°C for further future analyses. Of the adipose tissue deposits, only retroperitoneal adipose tissue was harvested and weighed because of its more distinct location and uniform composition compared to visceral (i.e., omental) and/or ovarian adipose tissue deposits. Plasma (EDTA) was separated from the whole blood via centrifugation after 15 min incubation at RT (1,500 g, 10 min at RT) and stored as 200 μL aliquots in −80°C.

#### 2.10.1 Plasma HDL-C level

We (Karvinen et al., 2019) and others have previously shown that estrogen deficiency caused by menopause is associated with increased high-density lipoprotein cholesterol (HDL-C) levels in women (Pansini et al., 1993; Mandrup et al., 2017). To study whether OVX causes similar effect, the HDL-C level was measured from the plasma of representative subsets of the groups (*n* = 16) using an automated analyzer (Indiko Plus, Thermo Fisher, US).

#### 2.10.2 Protein analysis from skeletal muscle and adipose tissues via Western blot

Snap frozen gastrocnemius muscle and retroperitoneal adipose tissue were used for Western blot analysis as described previously (Karvinen et al., 2016). The tissue sample was dissolved in ice-cold buffer [20 mM HEPES [pH 7.4], 1 mM EDTA, 5 mM EGTA, 10 mM MgCl_2_, 100 mM, β-glycerophosphate, 1 mM Na_3_VO_4_, 2 mM DTT, 1% NP-40 (nonyl phenoxypolyethoxylethanol), 0.2% sodium deoxycholate, and 3% protease and phosphatase inhibitor cocktail (P 78443; Pierce, Rockford, IL)] and homogenized using a stainless steel bead in TissueLyser II (Qiagen, Germany) for 2 min at 30 Hz. Samples were mixed for 30 min in end-over-end rotation at +4°C. The tissue homogenate was then centrifuged at 10,000 x g for 10 min at +4°C. Total protein content was determined using the bicinchoninic acid protein assay (Pierce Biotechnology, Rockford, IL) with an automated KoneLab instrument (Thermo Scientific, Vantaa, Finland).

Aliquots of tissue homogenate were solubilized in Laemmli sample buffer and heated at 95°C to denaturate proteins, except for detecting mitochondrial protein complexes via Total OXPHOS Cocktail, when samples were heated at 37°C. Samples containing 30 μg of total protein were separated by SDS-PAGE for 30 min at 270 V using 4–20% gradient gels on Criterion electrophoresis cell (Bio-Rad Laboratories, Richmond, CA). Proteins were transferred to nitrocellulose membranes, which were blocked in blocking buffer (Licor, Cat no 927–70001) for 2 h and then incubated overnight at +4°C with primary antibodies in 1:1 TBS and blocking buffer to analyze the content of pRLC (1:1000; ab2480, Abcam), Total OXPHOS Cocktail (OXPHOS, 1:1000; ab110413; Abcam), UCP1 (1:1000; ab10983, Abcam), UCP2 (1,500; ab67241 Abcam), and UCP3 (1,1,000; ab3477 Abcam). The proteins pRLC, OXPHOS, UCP2, and UCP3 were measured from gastrocnemius muscle and the proteins OXPHOS, UCP1, and UCP2 were measured from retroperitoneal adipose tissue.

After the primary antibody incubation, membranes were washed in TBS-Tween (TBS-T, 0.1%), and then incubated with a suitable secondary antibody diluted in TBS-T with 1:1 blocking buffer for 1 h followed by washing in TBS-T. Proteins were visualized using fluorescent secondary antibodies and quantified using ChemiDoc MP device (Bio-Rad Laboratories, Hercules, CA, United States) and quantified with Image Lab software (version 6.0; Bio-Rad Laboratories, Hercules, CA, United States). First, the target and total protein and levels were normalized to the membrane average to eliminate differences in signal intensity between the membranes. Thereafter, target protein levels were normalized to corresponding total protein amount (full lane) and results are shown in arbitrary units (AU).

### 2.11 Statistics

The summary statistics are presented as group means with standard deviations. The normality of variables was assessed using the Shapiro–Wilk test followed by Levene’s test for examining the equality of the variances. When the normality criteria were met, the differences between groups were examined using Student’s *t*-test and the difference between PRE and POST measurements using Student’s paired *t*-test. OVX and sham groups exhibited different variances in RER and thus, for this variable, comparisons were performed using Welch’s *t*-test. When the normality criteria were not fulfilled, the differences between groups were examined using Mann–Whitney U-test and the difference between PRE and POST measurements using Wilcoxon signed-rank test. The main effects of OVX, acute exercise and their interaction (OVX × Acute exercise) were determined using two-way analysis of variance (ANOVA). Energy expenditure is highly dependent on body mass and regression-based methods are needed particularly when comparing groups with differing masses (Müller et al., 2021). Therefore, changes in energy expenditure outcomes between groups were also compared using linear mixed-effect models (*nlme* package) with animal identification as the random effect, group × time interaction as the explanatory variable, and body mass as a covariate. For the analysis of changes in 24 h energy expenditure, a separate model also included energy intake and spontaneous activity as covariates. Correlation analyses were performed and visualized using *metan* package. In all analyses, a *p*-value ≤0.05 was considered to indicate statistical significance. Linear mixed-effect models and correlation analysis were generated using R version 4.3.1.

## 3 Results

### 3.1 OVX led to increased body mass and decreased maximal running capacity

**Figure 2:**
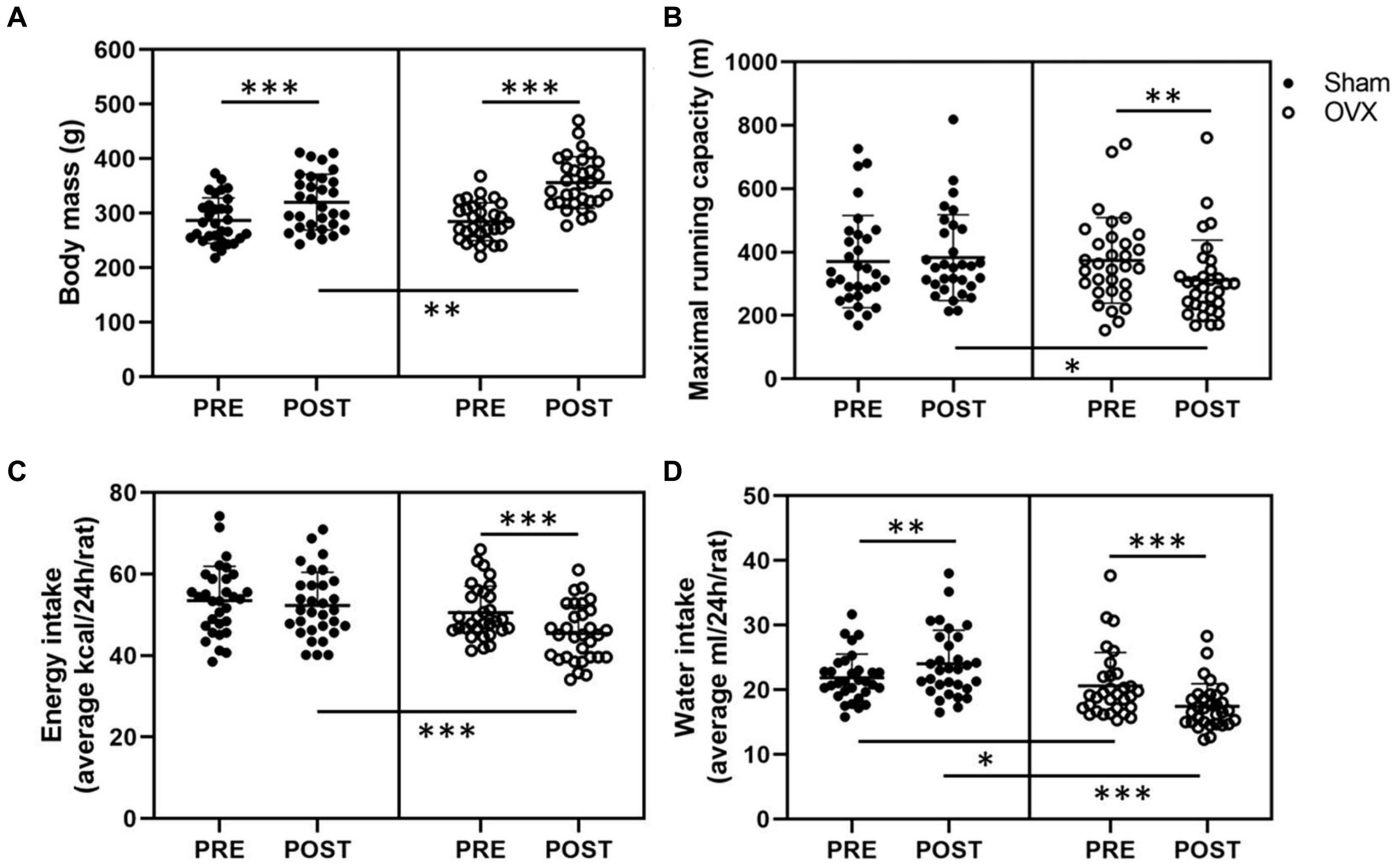
Body mass **(A)**, maximal running capacity **(B)**, energy intake **(C)**, and water intake **(D)** before (PRE) and after (POST) the sham/OVX surgeries. Figures show individual samples with mean and SD. ^*^p ≤ 0.050, ^**^p ≤ 0.010, and ^***^p ≤ 0.001.

Before the surgeries, the rats were divided into sham and OVX groups matched for body mass and maximal running capacity (*n* = 32/group). Accordingly, the sham and OVX groups did not differ in their body mass or maximal running capacity at PRE measurements (sham vs. OVX *p* ≥ 0.642, Figures 2A,B). In line with previous literature, OVX rats were heavier than sham rats at POST measurements (sham vs. OVX, *p* ≤ 0.01, Figure 2A). On average, sham group gained weight by 12% vs. 25% in the OVX group throughout the study (*p* ≤ 0.001, Figure 2A). The weekly body mass tracking revealed a significant difference in body mass between sham and OVX groups starting 3 weeks post-surgery (Supplementary Figure S1A).

Prior to the surgeries, the estrous cycle phase distribution in the whole study population during the best running trial was the following: proestrus 23%, estrus 23%, metestrus 16%, and diestrus 39%, indicating that maximal running capacity was not associated with higher estrogen level (i.e., proestrus). At POST measurements, OVX group had lower maximal running capacity than the sham group (sham vs. OVX *p* = 0.013, Figure 2B). On average OVX rats reduced their maximal running capacity by ~17% (*p* = 0.005, Figure 2B). There were no differences between the sham and OVX groups in work (J) performed during the maximal running tests in PRE or POST measurements [PRE sham: 300 (109), OVX: 295 (83); POST sham: 341 (107), OVX: 310 (95), *p* ≥ 0.245], yet within sham group the increase in work at POST vs. PRE measurements was significant (*p* = 0.008).

The sham and OVX groups did not differ in average daily energy intake (*p* = 0.174, Figure 2C) at PRE measurements, whereas at POST measurements, the average energy intake of OVX group had decreased (*p* < 0.001, Figure 2C). Rats in the sham group had higher water intake than rats in the OVX group both PRE and POST surgeries (*p* ≤ 0.025). Water intake post-surgery increased in the sham group and decreased in the OVX group (*p* ≤ 0.004, Figure 2D). The energy intake did not differ between sham and OVX groups in weeks 7–13 (*p* ≥ 0.139) while the OVX group had lower energy intake at 14 weeks (*p* = 0.011, Supplementary Figure S1B). Consistent with the water intake measured from individual rats, rats in the OVX group had lower water intake between weeks 7–14 (*p* ≤ 0.031, Supplementary Figure S1C).

### 3.2 OVX was associated with higher fat mass and higher HDL-C level

In addition to having higher body mass, the OVX group had higher retroperitoneal adipose tissue mass and lower liver mass compared with the sham group (*p* ≤ 0.005, Table 1). The uterine mass of rats in the sham group was larger compared with the OVX group, which affirms the success of the OVX surgeries (*p* ≤ 0.001, Table 1). Picture of representative uteri at the end of the study are shown in Supplementary Figure S2A. There were no differences in the individual muscle masses between the groups, yet OVX group had lower total muscle mass/body mass ratio compared with the sham group (*p* ≤ 0.001, Table 1).

**Table 1:** Body and tissue masses at the end of the study and blood parameters of sham and OVX rats (mean with SD).

	Sham	OVX	*p*
Tissue masses (g)	***n* = 29–30**	***n* = 31**	
Body mass	323 (55)	362 (50)	**0.005**
Heart	0.877 (0.120)	0.854 (0.143)	0.436
Liver	9.294 (1.274)	8.186 (1.411)	**0.002**
Retroperitoneal adipose tissue	5.917 (2.910)	9.946 (3.397)	**<0.001**
Uterine mass	0.726 (0.150)	0.137 (0.048)	**<0.001**
Muscle masses (g)			
^¤^Soleus	0.121 (0.019)	0.117 (0.017)	0.573
^¤^Plantaris	0.286 (0.039)	0.283 (0.026)	0.659
^¤^Gastrocnemius	1.526 (0.196)	1.542 (0.151)	0.737
^¤^EDL	0.134 (0.017)	0.138 (0.016)	0.415
^¤^TA	0.603 (0.076)	0.609 (0.067)	0.774
#Total muscle mass	2.670 (0.335)	2.688 (0.261)	0.690
#Total muscle mass/body mass	0.785 (0.218)	0.748 (0.058)	**0.001**
Blood parameters	***n* = 15–31**	***n* = 16–31**	
HDL-C	2.272 (0.468)	2.611 (0.305)	**0.022**
Fasting blood glucose (mmol/l)			
PRE	5.400 (0.640)	5.506 (0.651)	0.606
POST	5.206 (0.996)	5.152 (0.671)	0.607
Fasting blood insulin (μg/l)			
PRE	0.361 (0.121)	0.410 (0.185)	0.786
POST	0.374 (0.189)	0.686 (0.313)	**<0.001**
HOMA-IR			
PRE	0.830 (0.257)	0.938 (0.451)	0.759
POST	0.817 (0.448)	1.502 (0.776)	**<0.001**

^¤^Average of right and left muscle. ^#^Sum of average of right and left muscle masses of soleus, plantaris, gastrocnemius, EDL and TA. Significant p-values marked in bold.

The blood parameters revealed that OVX rats had higher plasma HDL-C level compared with sham rats (*p* = 0.022, Table 1). The fasting blood glucose did not differ between the sham and OVX groups PRE or POST surgeries (*p* ≥ 0.606, Table 1), yet OVX group had higher fasting insulin and HOMA-IR levels at POST measurements compared with sham group (*p* < 0.001).

At PRE measurements, rats allocated to the sham and OVX groups did not differ in energy expenditure, RER, spontaneous activity level, and energy or water intake (*p* ≥ 0.129, Table 2). Even though the OVX rats were heavier at POST measurements, their absolute energy expenditure was lower, particularly during dark hours (Table 2). When adjusted for body mass, 24 h energy expenditure decreased by −0.22 kcal/h (95% CI -0.30 to −0.15, *p* < 0.001) in the OVX group compared with the sham group. When examining the light and dark hours separately, energy expenditure during light hours decreased by −0.15 kcal/h (95% CI −0.22 to −0.08, *p* < 0.001) and during dark hours by −0.30 kcal/h (95% CI -0.40 to −0.20, *p* < 0.001) in the OVX group compared with the sham group. Furthermore, when adjusted for body mass, energy intake and spontaneous activity, 24 h energy expenditure decreased by −0.20 kcal/h (95% CI -0.28 to −0.12, *p* < 0.001) in the OVX group compared with the sham group. RER and spontaneous activity decreased in both groups during the study and were similar between groups at PRE and POST measurements (Table 2). Both energy and water intake decreased in the OVX group (*p* < 0.001, Table 2) and at POST measurements, the OVX group consumed less water than the sham group (*p* < 0.001, Table 2).

**Table 2:** Energy expenditure, RER, spontaneous activity, and energy and water intake of sham and OVX rats (mean with SD).

	PRE				POST				Change			
	SHAM	OVX	Difference	*p*-value	SHAM	OVX	Difference	*p*-value	SHAM	*p*-value	OVX	*p*-value
Energy expenditure (kcal/h)												
24 h	1.77 (0.19)	1.75 (0.19)	0.020	0.728	1.92 (0.17)	1.77 (0.16)	0.150	**< 0.001**	0.16	**< 0.001**	0.02	0.331
Light h	1.46 (0.14)	1.46 (0.16)	0.000	0.982	1.66 (0.13)	1.58 (0.15)	0.070	0.053	0.19	**< 0.001**	0.12	**< 0.001**
Dark h	2.07 (0.26)	2.03 (0.24)	0.040	0.580	2.19 (0.22)	1.96 (0.19)	0.230	**< 0.001**	0.12	**< 0.001**	−0.08	**0.018**
Respiratory exchange ratio (RER)												
24 h	0.87 (0.06)	0.89 (0.03)	0.020	0.168	0.83 (0.06)	0.84 (0.03)	0.000	0.884	−0.04	**0.003**	−0.05	**< 0.001**
Light h	0.84 (0.06)	0.85 (0.04)	−0.010	0.300	0.81 (0.06)	0.81 (0.03)	0.000	0.824	−0.03	**0.019**	−0.04	**< 0.001**
Dark h	0.90 (0.06)	0.93 (0.04)	−0.020	0.137	0.86 (0.07)	0.86 (0.03)	0.000	0.949	−0.05	**0.001**	−0.07	**< 0.001**
Spontaneous Activity (AU)												
24 h	0.057 (0.020)	0.053 (0.019)	0.003	0.459	0.042 (0.015)	0.045 (0.020)	−0.003	0.522	−0.015	**0.003**	−0.008	**0.029**
Light h	0.027 (0.012)	0.026 (0.011)	0.002	0.456	0.021 (0.010)	0.025 (0.012)	−0.003	0.247	−0.005	0.072	0	0.970
Dark h	0.086 (0.032)	0.081 (0.028)	0.006	0.496	0.062 (0.021)	0.065 (0.030)	−0.003	0.688	−0.024	**0.001**	−0.016	**0.004**
Energy intake (kcal)												
24 h	42.0 (13.2)	45.0 (11.9)	−3.0	0.361	41.1 (14.9)	36.5 (8.6)	4.6	0.150	−0.8	0.790	−8.5	**< 0.001**
Water intake (ml)												
24 h	21.9 (7.0)	19.3 (6.0)	2.6	0.129	23.1 (6.4)	14.1 (4.7)	9.1	**< 0.001**	1.3	0.362	−5.2	**< 0.001**

Significant p-values marked in bold.

### 3.3 Acute exercise was associated with low serotonin level in the hippocampus (HC) and high level in the nucleus accumbens (NA) only among OVX rats

Before the euthanasia, the rats in the sham and OVX groups were divided into control and maximal running test (max) subgroups matched for body mass and maximal running capacity (*n* = 15–16/group) (Figure 1B). Consistent with this, the groups (sham/control vs. sham/max and OVX/control vs. OVX/max) did not differ in their body mass or maximal running capacity (*p* ≥ 0.734, Supplementary Figures S3A,B), yet body mass was higher and maximal running distance lower in the OVX compared with sham at POST measurements (*p* ≤ 0.013, Supplementary Figures S3A,B). The running distance the rats performed right before euthanasia did not differ between the groups (sham/max vs. OVX/max, *p* = 0.299, Supplementary Figure S3C).

**Figure 3:**
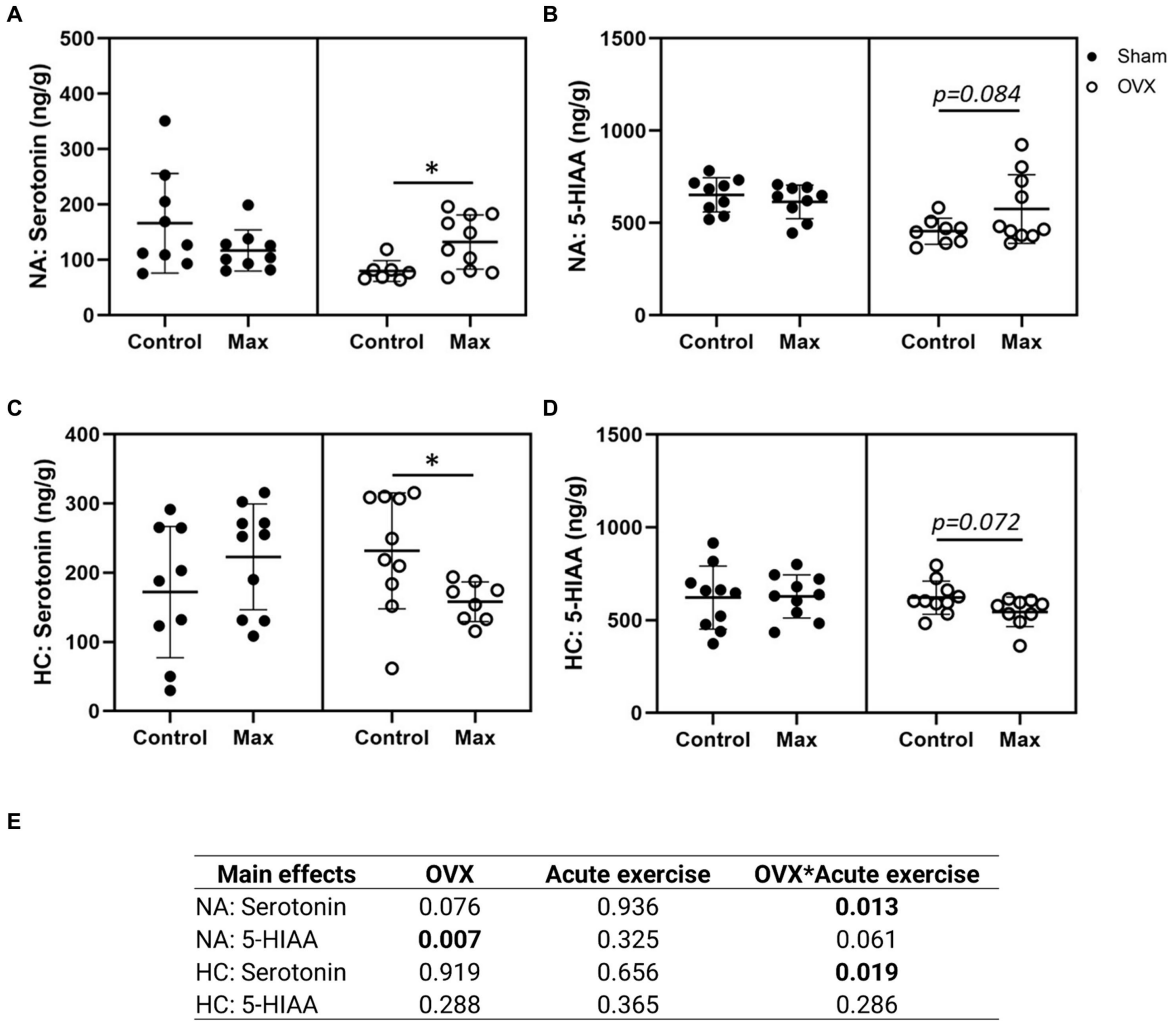
Serotonin **(A)** and serotonin metabolite 5-HIAA **(B)** in the nucleus accumbens (NA) and in the hippocampus (HC) (**C,D**). The main effects of OVX, acute exercise and their interaction on the measured neurochemical markers **(E)**. Figures show individual samples with mean and SD. ^*^p ≤ 0.050.

All seven measured neurochemical markers were detected in the NA (Figures 3A,B; Supplementary Figures S4A–E). OVX was associated with lower serotonin metabolite, 5-HIAA, level in NA compared to sham (*p* ≤ 0.007, Figure 3E). Interestingly, acute exercise was associated with an increase in NA serotonin level in OVX rats (*p* = 0.036), but not in sham rats (Figures 3A,E).

Of the seven measured neurochemical markers, four were detected in the HC: dopamine, serotonin, norepinephrine, and the serotonin metabolite 5-HIAA (Figures 3C,D; Supplementary Figures 4E,F). In contrast to NA, neither serotonin nor 5-HIAA levels were significantly affected by OVX in HC (Figures 3C,D). However, unlike the case with NA, acute exercise was associated with a reduction in HC serotonin level (*p* = 0.024, Figure 3C) in OVX animals only. Uncertainty remains whether acute exercise is also associated with elevated 5-HIAA levels in OVX animals (*p* = 0.084, Figure 3B).

We also examined the effects of OVX, acute exercise, and their interaction on serotonin/dopamine and serotonin/norepinephrine ratios but observed no significant effects (*p* ≥ 0.056, Supplementary Figure S5). The closest to a significant observation was the interaction of OVX and acute exercise in NA serotonin/dopamine ratio (*p* = 0.056, Supplementary Figure S5A).

Together with the significant interaction of OVX and acute exercise on serotonin in both NA and HC our results indicate that OVX rats have a distinct response to exercise in these two brain regions.

### 3.4 OVX was associated with lower UCP3 level in gastrocnemius muscle

**Figure 4:**
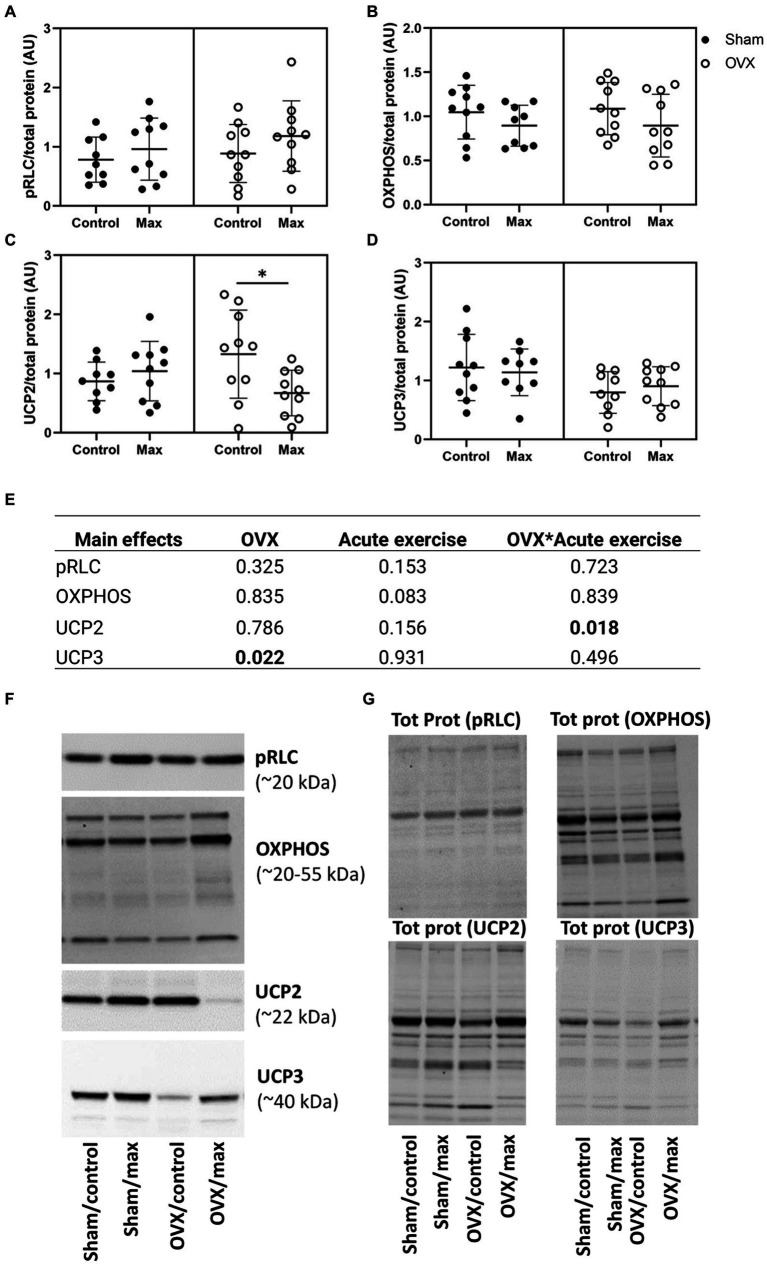
Protein levels of pRLC **(A)**, OXPHOS **(B)**, UCP2 **(C)**, and UCP3 **(D)** relative to total protein level in gastrocnemius muscle with representative blot images **(F)** and corresponding blot images of total protein content **(G)**. The main effects of OVX, acute exercise and their interaction on the measured proteins (**E**). Tot prot, total protein. Figure shows individual samples with mean and SD. ^*^p < 0.050.

There were no effects of OVX, acute exercise, or their interaction on pRLC level in the gastrocnemius muscle (*p* ≥ 0.153, Figure 4). However, OVX was associated with lower UCP3 level compared to sham (*p* = 0.022), which was not affected by exercise in either group. There was an interaction of OVX and acute exercise for UCP2 level in gastrocnemius level (*p* = 0.018) such that exercise reduced UCP2 in OVX rats only (*p* = 0.023, Figures 4C–E).

### 3.5 OVX did not affect the level of studied proteins in retroperitoneal adipose tissue

**Figure 5:**
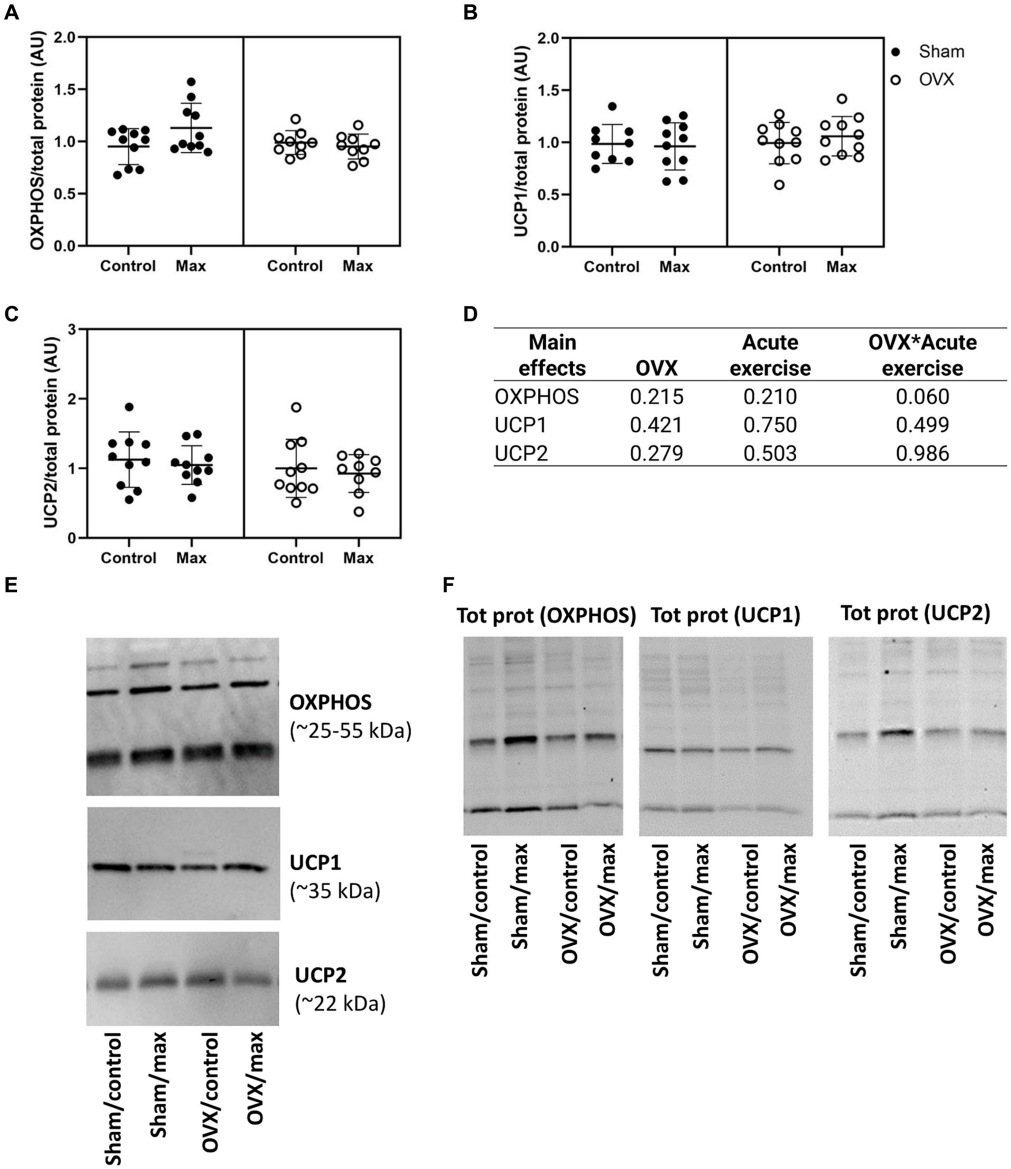
Protein levels of OXPHOS **(A)**, UCP1 **(B)**, and UCP2 **(C)** relative to total protein level in retroperitoneal adipose tissue with representative blot images **(E)** and corresponding blot images of total protein content **(F)**. The main effects of OVX, acute exercise and their interaction on the measured proteins (**D**). Figure shows individual samples with mean and SD. Tot prot, total protein.

We observed no effects of OVX, acute exercise or their interaction in the protein levels of OXPHOS, UCP1 or UCP2 in retroperitoneal adipose tissue (*p* = 0.060, Figure 5).

### 3.6 Maximal running capacity was positively associated with serotonin and 5-HIAA levels in the nucleus accumbens (NA)

To gain additional insight into the physiological relevance of OVX’s effects on brain region-specific responses to exercise, correlations among neurochemical markers and parameters of health, performance and metabolism were assessed.

**Figure 6:**
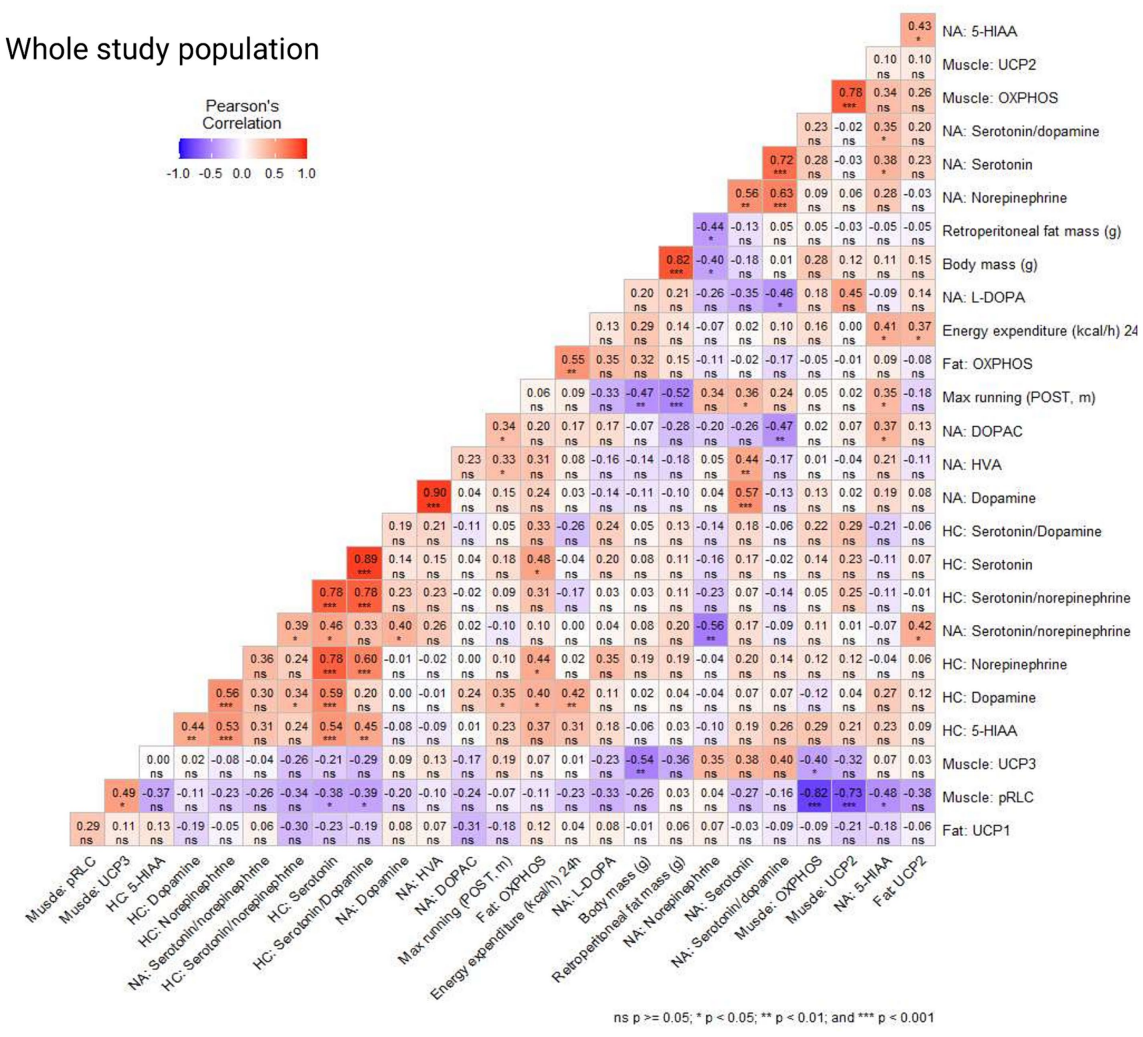
Correlation coefficients (Pearson’s correlation) in the whole study population of brain neurochemical markers in the nucleus accumbens (NA) and hippocampus (HC), protein levels in muscle (gastrocnemius), adipose tissue (retroperitoneal deposit), body mass, retroperitoneal fat mass, maximal running capacity (POST, END), and 24 h energy expenditure (kcal/h).

When examining the correlations within the whole study population, we observed a positive correlation between maximal running capacity (POST) and dopamine level in the HC, and serotonin, DOPAC, 5-HIAA, and HVA levels in the NA (*p* ≤ 0.048, Figure 6). Also, muscle pRLC protein level had a positive correlation with maximal running capacity examined immediately prior to euthanasia (END, *p* = 0.017). Norepinephrine level in the NA was negatively correlated with body mass and retroperitoneal adipose tissue mass (*p* ≤ 0.009, Figure 6), while 24 h energy expenditure (kcal/h) had a positive correlation with dopamine level in HC and 5-HIAA level in NA as well as OXPHOS and UCP2 protein levels measured from the retroperitoneal adipose tissue (*p* ≤ 0.050, Figure 6). Interestingly, L-DOPA level in the NA had a positive correlation with retroperitoneal fat mass (*p* = 0.044), but not with body mass (*p* = 0.124, Figure 6). In addition, adipose tissue OXPHOS protein level had positive correlations with dopamine, serotonin and norepinephrine levels in the HC (*p* ≤ 0.037, Figure 6). Expectedly, body mass and retroperitoneal fat mass had a positive correlation (*p* < 0.001). Additionally, maximal running capacity was negatively correlated with body mass and retroperitoneal fat mass (*p* ≤ 0.002, Figure 6). We observed no significant correlation between the maximal running capacity results (POST, END) and serotonin/dopamine ratios in NA or HC (*p* ≥ 0.160, Figure 6). Serotonin and its metabolite 5-HIAA had a positive correlation both in NA and HC (*p* ≤ 0.005, Figure 6), indicating robust measurement of the brain neurochemical markers.

We examined the same correlations in the following study populations separately: sham, OVX, control (no acute exercise) and max (acute exercise) and observed minor differences between the study populations (Supplementary Figures S6–S9). In the sham group, body mass had a positive correlation with OXPHOS protein level measured from gastrocnemius muscle (*p* ≤ 0.016, Supplementary Figure S6), while in the OVX group body mass had a positive correlation with OXPHOS and UCP2 protein levels measured from retroperitoneal adipose tissue (*p* ≤ 0.032, Supplementary Figure S7). Interestingly, pRLC level in the muscle was positively correlated with maximal running capacity (END) in the sham group, but not in OVX group (*p* = 0.036 and *p* = 0.145, respectively, Supplementary Figure S6). OVX group had a negative correlation between maximal running capacity (END) and serotonin/dopamine ratio in HC (*p* = 0.011, Supplementary Figure S7). In the control group there was a positive correlation between maximal running capacity (POST) and serotonin/dopamine ratio in NA (*p* = 0.036, Supplementary Figure S8). In the max (acute exercise) group the maximal running capacity (END) had a negative correlation with L-DOPA level in NA (*p* = 0.021) and a strong positive correlation with max running capacity (POST). This suggests that the maximal running tests performed immediately before euthanasia had similar results with maximal running tests performed at time point POST (*p* = 0.001, Supplementary Figure S9).

## 4 Discussion

In this study, age-appropriate adult female rats were ovariectomized (OVX) or underwent sham operations to assess the effect of age-related ovarian hormone loss (i.e., mimicking menopause in women) on metabolism and body composition, as well as skeletal muscle, adipose tissue, and brain region-specific responses to an acute exercise stimulus. We show that in addition to causing weight gain due to reduced energy expenditure, OVX led to reduced maximal running capacity despite no changes in lower limb muscle mass or the pRLC level in the gastrocnemius muscle. Above all, we report for the first time that OVX affected the brain’s response to exercise in a brain region-specific manner. These findings may offer novel mechanistic insight into the role of specific brain regions in mediating OVX-induced changes in one’s willingness to run.

### 4.1 OVX increased adiposity and reduced energy expenditure

In line with previous literature, OVX led to increased adiposity, and when measured ~8 weeks post-surgeries, a reduced total energy expenditure. Unsurprisingly, OVX rats were heavier than sham-operated rats (Chen and Heiman, 2001; Asarian and Geary, 2002; de Souza et al., 2019). However, this occurred in the absence of significant differences in spontaneous physical activity, which appears contradictory to other published works (Rodier, 1971; Kadi et al., 2002; Hertrampf et al., 2006). In the present study, the difference in body mass between the groups became apparent by three weeks post-surgeries. Plausible explanations are increased energy intake, decreased spontaneous activity (i.e., activity-related energy expenditure) and/or decreased basal energy expenditure.

Our data support reduced energy expenditure, and not increased energy intake or decreased spontaneous activity, as the main cause of adiposity increase. While the sham and OVX groups did not differ in daily energy intake prior to surgeries, energy intake of the OVX group declined after surgery despite weight gain. In spite of our findings, we cannot entirely rule out the possibility that OVX-induced weight gain may be due to increased energy intake, as previous studies have observed an increase in energy intake following OVX in rats (Chen and Heiman, 2001; Asarian and Geary, 2002; de Souza et al., 2019). However, in these studies there was only a short-term follow-up (~1-month post-surgery) on energy intake. Hence, it is possible that the energy intake ultimately declined in those studies as well. Indeed, McElroy and Wade (1987) reported that OVX increased energy intake in a transient manner such that intake increased during the first 5 weeks after the surgery but normalized to the level of controls during weeks 6–10 (McElroy and Wade, 1987). Importantly, despite the similar energy intake, the body weights of the OVX rats remained 12–16% above sham group, similar to what we observed in the current study. Serotonergic signaling contributes to the metabolic signals that facilitate the ability to suppress food intake, while disturbed serotonergic signaling is associated with obesity (van Galen et al., 2021). However, unraveling the serotonergic regulation of feed intake is complex, as the effects of signaling in different brain regions depend on the regional expression of serotonin receptor subtypes as well as downstream effects through connections to other brain regions (van Galen et al., 2021). In turn, it is well established that serotonin can affect physical activity, but the direction of the response appears to be both dose and time dependent (Shimizu et al., 1991; Chaouloff, 1997). In our study, OVX was associated with lower 5-HIAA level in NA, yet we observed no increase in the feed intake or reduction in spontaneous activity following OVX, substantiating that reduced energy expenditure is the main cause of adiposity increase.

Regarding energy expenditure, we observed no significant differences in spontaneous activity levels between the groups during the 24 h metabolic measurements before or after the surgeries. This is contrary to several other studies that have reported substantial decreases in voluntary wheel running activity in rats after OVX (Rodier, 1971; Kadi et al., 2002; Hertrampf et al., 2006). A decrease in voluntary spontaneous activity in OVX rats compared with sham rats both immediately (Giles et al., 2010) and 6 weeks after the surgeries (Izumo et al., 2012) have been reported. The 24 h assessment period used in the present study may have been too short to detect differences, since some studies use longer (e.g., 72-h or greater) assessment period. We did, however, detect differences in energy expenditure, which decreased in the OVX group. This was present even after controlling for body mass, energy intake and spontaneous activity, providing robust evidence that the loss of ovarian hormone stimulation decreased basal energy expenditure. An earlier study observed an increase in energy intake during the early period of OVX-induced weight gain (within 3 weeks of surgery), while only a minor change in total energy expenditure was detected (Giles et al., 2010). Studies in mice also support that OVX decreases energy expenditure without altering energy intake (Rogers et al., 2009). It is thus likely that OVX-mediated reduction in energy expenditure occurs more slowly and may not be detected until several weeks following surgery.

Mechanistically, decreased fat oxidation has been proposed to explain menopause-associated weight gain (Lovejoy et al., 2008). However, we found no differences in respiratory exchange ratio (RER) values, as an index of relative fat oxidation, between the sham and OVX groups. This finding is expected because the animals were in energy balance and were fed same the chow. Based on laws of conservation of energy and mass, RER between groups will be identical in this case (Péronnet and Haman, 2019). OVX did not alter substrate partitioning in the present study, and the reduction in energy expenditure might not be explained by decreases in fat oxidation. It is also possible that our system was not sensitive enough to detect subtle differences in RER over the 24 h time period of assessment. Regardless, our results suggest a decrease in basal energy expenditure may contribute to the increased body mass caused by OVX. The exact mechanisms are somewhat vague, and we are also uncertain of how well these data translate to humans, since whether menopause directly leads to reduced basal energy expenditure in middle-aged women remains unknown (Karppinen et al., 2023).

Regarding mechanisms by which loss of ovarian hormones in rodents or women results in changes in energy expenditure, there are a growing number of studies suggesting that estrogen may affect uncoupling proteins (UCP), which dissipate energy at the cellular level by uncoupling oxygen utilization from ATP production (Rousset et al., 2004). Indeed, estrogen-sufficient rodents and humans have been shown to have greater UCP1 expression in adipose tissue compared to age-matched males (Moschinger et al., 2019; Keuper and Jastroch, 2021; Queathem et al., 2021). Moreover, a reduction in UCP1 expression is associated with a reduction in basal metabolism (Clookey et al., 2018). In the present study, the rats were housed below thermoneutrality, which likely affects the level of UCPs; we measured UCPs 1, 2, and 3 in skeletal muscle and adipose tissue.

Compared to the sham group housed under the same temperature conditions, OVX did not appear to significantly affect UCP1 or UCP2, yet was associated with lower UCP3 protein content in skeletal muscle. UCP3 is the main uncoupling protein expressed in skeletal muscle and is known to play a significant role in non-shivering thermogenesis (Boss et al., 1997; Rousset et al., 2004). Moreover, mice overexpressing UCP3 in skeletal muscle weigh less, have a decreased amount of adipose tissue, are protected from fat-induced insulin resistance, and have increased resting oxygen consumption (Clapham et al., 2000; Choi et al., 2007). Thus, the lower expression of UCP3 in OVX rats may have contributed to their increased fat mass and decreased energy expenditure. Indeed, skeletal muscle UCP3 was negatively associated with body mass. Although not significantly affected by OVX, the adipose tissue levels of UCP2 and mitochondrial OXPHOS proteins were positively associated with energy expenditure. This supports the growing body of literature suggesting that white adipose tissue contributes significantly to resting energy expenditure (Heymsfield et al., 2019, 2021), especially among females who have greater relative adiposity. While these data are intriguing and warrant further study, it is likely that a variety of factors (e.g., rodent line/strain, age at OVX, type of diet, etc.) influence the changes in energy balance and energy expenditure that occur with the loss of ovarian hormones. Future studies should carefully control for such factors to obtain a better understanding of their interplay.

### 4.2 OVX increased adiposity and caused metabolic dysfunction

In addition to having higher body mass, the OVX group had higher retroperitoneal adipose tissue mass (i.e., a visceral depot) and lower liver mass compared with the sham group, in agreement with previous studies (Babaei et al., 2010; de Souza et al., 2019). The increase in body fat also concurs with findings from human studies done by our group (Juppi et al., 2022) and others (Lovejoy et al., 2008; Greendale et al., 2019), demonstrating an accelerated increase in adiposity during menopause. Estrogen deficiency with a concurrent increase in adipose tissue may lead to a greater risk of metabolic diseases, such as type 2 diabetes (Lee et al., 2021; Lambrinoudaki et al., 2022).

Some studies have observed a significant increase in fasting blood glucose after OVX (Tawfik et al., 2015; Min et al., 2018), conforming with the observation that estrogen deficiency results in declined insulin-stimulated glucose disposal (Livingstone and Collison, 2002). We did not observe a difference in fasting glucose levels, but the OVX group had higher fasting insulin levels as well as higher HOMA-IR than the sham group, indicating impaired insulin action. This observation corresponds to previous findings showing exacerbation of insulin resistance with estrogen deficiency (Livingstone and Collison, 2002; Tawfik et al., 2015).

In the present study, the OVX group had higher HDL-C compared with the sham group, which has been observed before (Bae et al., 2011). It has been suggested that the atheroprotective effect of HDL may be weaker in women after menopause (Woodard et al., 2011; El Khoudary et al., 2018). Previously we (Karvinen et al., 2019) and others (Woodard et al., 2011; Fernandez and Murillo, 2016) have reported a higher HDL-C level following menopause in women (Woodard et al., 2011; El Khoudary et al., 2018). This previously observed increase in HDL-C levels following menopause is replicated in our present animal study and thus validating prior findings.

### 4.3 OVX reduced maximal running capacity

Expectedly, OVX reduced maximal running capacity. Firstly, we ruled out an effect of current circulating estrogen level on running by examining the effect of estrous cycle phase on the best running trial. Similar to previously published data in women and rodents (Aguiar et al., 2018; Gordon et al., 2018) observed no association between the highest estrogen phase (i.e., proestrus) and best running trial among sham rats. However, as predicted, long-term estrogen deficiency caused by OVX led to lower maximal running capacity compared with the sham treatment. We then examined two possible contributors to the observed decrease in running capacity: phosphorylation of regulatory light chain of myosin (pRLC) protein content in skeletal muscle (i.e., a physiological change), and brain neurochemical markers (i.e., a neurochemical change).

It has previously been shown that estradiol modulates RLC activity in skeletal muscle (EDL) in mice (Lai et al., 2016). Specifically, OVX-operated C57BL/6 female mice had lower pRLC level in EDL muscle compared with sham-operated mice (Lai et al., 2016). However, we observed no differences between sham and OVX groups in the pRLC level in gastrocnemius muscle. Mouse EDL muscle is comprised of fast type (type II) muscle fibers (<1% type I fibers) (Augusto et al., 2004), whereas rat gastrocnemius muscle comprises of both fast and slow type (type I) muscle fibers (~10% type I fibers) (Bär and Pette, 1988). Thus, the difference in the muscle fiber type studied may partly explain the observed difference in the results. However, with regards to running, the gastrocnemius muscle is the largest lower limb muscle contributing to locomotion. Therefore, RLC phosphorylation might not be the key contributor to the observed decline in maximal running performance in the present study. We did find that pRLC protein level was positively associated with maximal running capacity (END), yet this observation was true only for the sham group. These results are in support of exercise stimulating the activation of RLC in skeletal muscle (Rayment et al., 1993; Del Coso et al., 2016) and suggest that OVX may affect the activation of RLC in response to an acute exercise stimulus. It may be that the increased fat mass of the OVX group (i.e., lower muscle mass relative to body weight) contributed to the reduced maximal running capacity, an idea supported by the fact that the total workload during the maximal running test was not significantly different in the sham and OVX groups in the POST measurements despite the lower running distance achieved by the OVX group. Indeed, it has been observed in humans that excess body weight can influence distance run performance (Cureton et al., 1978).

### 4.4 OVX affected the acute response to exercise differently across brain regions

Regarding brain region specific findings, OVX was associated with lower serotonin metabolite 5-hydroxyindoleacetic acid (5-HIAA) level in brain NA, in accordance with earlier works (Wissink et al., 2001; Rybaczyk et al., 2005). As in the case of energy intake, the level of neurochemical markers following OVX may change in a time-dependent manner. For example, a recent study showed that in mice, OVX was associated with reduced hippocampal 5-HIAA at 1 week, but not 6 weeks after the surgeries (Long et al., 2018). However, another study in mice observed that 5-HIAA levels in HC remained reduced even after 28 weeks following OVX (Heikkinen et al., 2002). In our study (~16 weeks post-OVX) we also observed a lower level of 5-HIAA following OVX, but this was true in the NA, and not in the HC brain region.

Contrary to previous literature, our results show that acute exercise had no effect on the level of serotonin in the sham group, while in OVX group it did affect brain serotonin levels, albeit in a brain region-specific manner. Based on prior research, exercise, particularly acute bouts, increases the serotonin and 5-HIAA levels in the whole brain as well as the HC [for a review, please see Meeusen and De Meirleir (1995)], while no data are available specific to the NA. To our knowledge, our study is the first to show that acute exercise is associated with higher serotonin levels in the NA only in the OVX group. We speculate that this response observed in the OVX group may indicate a compensatory mechanism caused by the serotonin deficiency in NA associated with the loss of ovarian hormones. Noteworthily, exercise had the opposite effect on serotonin levels in HC for the OVX animals. Since serotonin is suggested to modulate fatigue upon prolonged exercise, the difference in serotonin response to exercise may contribute to the animals’ experienced exhaustion during maximal running tests (Meeusen and De Meirleir, 1995).

Brain levels of serotonin, dopamine, and norepinephrine have all been linked to fatigue, thereby regulating physical performance (Cordeiro et al., 2017). Animal experiments indicate that increased serotonin reduces performance (Soares et al., 2003; Cordeiro et al., 2014), while increased dopamine and norepinephrine increases performance (Balthazar et al., 2009; Klass et al., 2016). Due to their interaction, serotonin/dopamine and possibly serotonin/norepinephrine ratio have been suggested to be more relevant for determining fatigue than analyzing only one neurochemical marker. However, we did not observe an effect of OVX, acute exercise or their interaction in serotonin/dopamine or serotonin/norepinephrine ratios in the NA or HC. Also, when looking at the whole study population, we observed no significant correlation between the maximal running capacity results (POST, END) and serotonin/dopamine ratios in the two brain regions. However, consistent with previous observations, among the OVX group, there was a negative correlation between maximal running capacity (END) and serotonin/dopamine ratio in the HC.

We noted a positive association between dopamine level in HC and maximal running capacity (POST) which has been shown previously (Park et al., 2016). In addition, HC dopamine level had a positive association with energy expenditure and markers of mitochondrial content (OXPHOS) in the retroperitoneal adipose tissue, which we believe to be the first report of a relationship between adipose tissue metabolism and HC levels of dopamine. Another relationship between brain neurotransmitter levels and body mass/ adiposity was observed such that norepinephrine level in the NA was negatively associated with body mass and retroperitoneal adipose tissue mass. Given that brain-derived norepinephrine stimulates UCPs, these findings do provocatively suggest that OVX-mediated changes in brain neurochemical levels may contribute to changes in basal energy expenditure, a hypothesis that was not tested in the present study yet warrants further study.

### 4.5 Strengths and limitations

This study had many strengths but was not free of limitations that should be considered. The present study was designed to investigate the effects of OVX on maximal running capacity in adult female rats. In OVX literature, an extensive disagreement exists as to the ideal age at which rats should undergo the OVX surgery in order to mimic human menopause. Many studies consider animals adult once the animal is sexually mature, which does not correspond to the age when the animal is fully grown, relative to humans. Hence one key strength of the current study is that the animals were fully grown adults (~7 months old) before undergoing surgery. The second strength of the study is the implementation of allocating animals into the four study groups matched for body weight and maximal running capacity. Moreover, there were no differences observed in energy expenditure or spontaneous activity between the sham and OVX groups at baseline. A limitation of the current study is that the energy intake was not followed immediately after the OVX surgeries, where there may have been a transient increase in the energy intake of OVX rats. However, the current study’s focus was the long-term effects of OVX and the acute effect of exercise. Since this study focused on neurochemical markers, we were unable to carry out thorough analysis of skeletal muscle, such as measuring other phosphoproteomic changes besides pRLC, which could have contributed to muscle function in the present study. In addition, although our study allowed sufficient time (~3 months) for estrogen deficiency to impact tissue protein levels, the period after acute exercise might not be optimal for detecting immediate changes in protein synthesis starting from transcription. Since the brain samples were collected as whole tissue samples, we are unable to differentiate whether the origin of neurotransmitters is intracellular or extracellular. As a result, the measured levels may include neurochemicals released into the extracellular space. Future studies with methodologies offering better temporal resolution are needed to address the possible functional consequences of the observed changes. It is also worth noting that OVX surgery causes a rapid decrease of systemic estrogens, and hence cannot be considered as an optimal model of human menopausal transition. Yet the long-term effects that OVX results in can represent some of the phenomena that takes place in women at the postmenopausal stage, such as increased fat mass and HDL-C levels, as reflected in our results.

### 4.6 Conclusions and future directions

In the adult female rats studied herein, OVX effectively mimicked menopause, as indicated by an increase in body mass during follow-up and higher fat mass, and HDL-C levels compared with the sham group. The changes in body mass and composition seemed to be driven by a decrease in basal energy expenditure. We report that OVX reduced maximal running capacity, but this was not accompanied by changes in muscle mass or pRLC in the gastrocnemius muscle. OVX was, however, associated with significantly lower brain serotonin metabolite 5-HIAA levels, confirming prior work by other groups. We extend those observations, demonstrating that the serotonin response to acute exercise differed in the two brain regions studied, HC and NA. These differences in the brain neurochemical response to an acute bout of exercise may be mechanistically related to the known OVX-associated changes in running behavior. These changes may potentially play a role in the reduced maximal running capacity, as well as the OVX-induced increases in body and fat mass. The relationship between neurochemical changes and whole-body energy expenditure may explain these findings. In summary, our findings provide a novel insight into understanding of how OVX impacts exercise motivation by introducing a possible underlying mechanism.

## Data availability statement

The original contributions presented in the study are included in the article/Supplementary material, further inquiries can be directed to the corresponding author.

## Ethics statement

The animal study was approved by National Project Authorization Board (ELLA, Finland, permit number ESAVI/4209/2021). The study was conducted in accordance with the local legislation and institutional requirements.

## Author contributions

EL: Writing – review & editing, Writing – original draft, Visualization, Methodology, Formal analysis. TN: Writing – review & editing, Writing – original draft, Methodology, Conceptualization. LY-O: Writing – review & editing, Methodology. AJ: Writing – review & editing, Methodology. JK: Writing – review & editing, Visualization, Formal analysis. VV-P: Writing – review & editing, Conceptualization. AL: Writing – review & editing, Methodology. SK: Writing – review & editing, Writing – original draft, Visualization, Validation, Supervision, Resources, Project administration, Methodology, Investigation, Funding acquisition, Formal analysis, Data curation, Conceptualization.

## References

[ref1] Aguiar A. S. Speck A. E. Amaral I. M. Canas P. M. Cunha R. A. (2018). The exercise sex gap and the impact of the estrous cycle on exercise performance in mice. Sci. Rep. 8:10742. doi: 10.1038/s41598-018-29050-0, PMID: 30013130 PMC6048134

[ref2] Alexander P. Vsevolod L. Natalia M. Pavel M. (2021). Effect of hindlimb unloading on recruitment of gastrocnemius medialis muscle during treadmill locomotion in rats. Exp. Brain Res. 239, 2793–2801. doi: 10.1007/s00221-021-06167-9, PMID: 34247266

[ref3] Asarian L. Geary N. (2002). Cyclic estradiol treatment normalizes body weight and restores physiological patterns of spontaneous feeding and sexual receptivity in ovariectomized rats. Horm. Behav. 42, 461–471. doi: 10.1006/hbeh.2002.1835, PMID: 12488112

[ref4] Augusto V. Padovani C. R. Campos G. E. R. (2004). Skeletal muscle fiber types in C57BL6J mice. Braz. J. Morphol. Sci. 21, 89–94.

[ref5] Babaei P. Mehdizadeh R. Ansar M. M. Damirchi A. (2010). Effects of ovariectomy and estrogen replacement therapy on visceral adipose tissue and serum adiponectin levels in rats. Menopause Int. 16, 100–104. doi: 10.1258/mi.2010.010028, PMID: 20956683

[ref6] Bae Y.-J. Choi M.-K. Kim M.-H. (2011). Manganese supplementation reduces the blood cholesterol levels in ca-deficient ovariectomized rats. Biol. Trace Elem. Res. 141, 224–231. doi: 10.1007/s12011-010-8714-1, PMID: 20455030

[ref7] Balthazar C. H. Leite L. H. R. Rodrigues A. G. Coimbra C. C. (2009). Performance-enhancing and thermoregulatory effects of intracerebroventricular dopamine in running rats. Pharmacol. Biochem. Behav. 93, 465–469. doi: 10.1016/j.pbb.2009.06.009, PMID: 19549536

[ref8] Bär A. Pette D. (1988). Three fast myosin heavy chains in adult rat skeletal muscle. FEBS Lett. 235, 153–155. doi: 10.1016/0014-5793(88)81253-5 3402594

[ref9] Behall K. M. Howe J. C. Martel G. Scott W. H. Dooly C. R. (2003). Comparison of resistive to aerobic exercise training on cardiovascular risk factors of sedentary, overweight premenopausal and postmenopausal women. Nutr. Res. 23, 607–619. doi: 10.1016/S0271-5317(03)00015-0

[ref10] Boss O. Samec S. Paoloni-Giacobino A. Rossier C. Dulloo A. Seydoux J.. (1997). Uncoupling protein-3: a new member of the mitochondrial carrier family with tissue-specific expression. FEBS Lett. 408, 39–42. doi: 10.1016/s0014-5793(97)00384-0, PMID: 9180264

[ref11] Cacho J. Sevillano J. de Castro J. Herrera E. Ramos M. P. (2008). Validation of simple indexes to assess insulin sensitivity during pregnancy in Wistar and Sprague-Dawley rats. Am. J. Physiol. Endocrinol. Metab. 295, E1269–E1276. doi: 10.1152/ajpendo.90207.2008, PMID: 18796548

[ref12] Chaouloff F. (1997). Effects of acute physical exercise on central serotonergic systems. Med. Sci. Sports Exerc. 29, 58–62. doi: 10.1097/00005768-199701000-00009, PMID: 9000156

[ref13] Chen Y. Heiman M. L. (2001). Increased weight gain after ovariectomy is not a consequence of leptin resistance. Am. J. Physiol. Endocrinol. Metab. 280, E315–E322. doi: 10.1152/ajpendo.2001.280.2.E315 11158936

[ref14] Chen W. Li J. Liu J. Wang D. Hou L. (2019). Aerobic exercise improves food reward Systems in Obese Rats via insulin signaling regulation of dopamine levels in the nucleus Accumbens. ACS Chem. Neurosci. 10, 2801–2808. doi: 10.1021/acschemneuro.9b00022, PMID: 31009571

[ref15] Chen H. Perez J. N. Constantopoulos E. McKee L. Regan J. Hoyer P. B.. (2014). A method to study the impact of chemically-induced ovarian failure on exercise capacity and cardiac adaptation in mice. J. Vis. Exp. 86:51083. doi: 10.3791/51083-v PMC4164028 24747886

[ref16] Choi C. S. Fillmore J. J. Kim J. K. Liu Z.-X. Kim S. Collier E. F.. (2007). Overexpression of uncoupling protein 3 in skeletal muscle protects against fat-induced insulin resistance. J. Clin. Invest. 117, 1995–2003. doi: 10.1172/JCI13579, PMID: 17571165 PMC1888566

[ref17] Clapham J. C. Arch J. R. Chapman H. Haynes A. Lister C. Moore G. B.. (2000). Mice overexpressing human uncoupling protein-3 in skeletal muscle are hyperphagic and lean. Nature 406, 415–418. doi: 10.1038/35019082, PMID: 10935638

[ref18] Clookey S. L. Welly R. J. Zidon T. M. Gastecki M. L. Woodford M. L. Grunewald Z. I.. (2018). Increased susceptibility to OVX-associated metabolic dysfunction in UCP1-null mice. J. Endocrinol. 239, 107–120. doi: 10.1530/JOE-18-0139, PMID: 30089681 PMC7340174

[ref19] Cordeiro L. M. S. Guimarães J. B. Wanner S. P. La Guardia R. B. Miranda R. M. Marubayashi U.. (2014). Inhibition of tryptophan hydroxylase abolishes fatigue induced by central tryptophan in exercising rats. Scand. J. Med. Sci. Sports 24, 80–88. doi: 10.1111/j.1600-0838.2012.01464.x, PMID: 22540893

[ref20] Cordeiro L. M. S. Rabelo P. C. R. Moraes M. M. Teixeira-Coelho F. Coimbra C. C. Wanner S. P.. (2017). Physical exercise-induced fatigue: the role of serotonergic and dopaminergic systems. Braz. J. Med. Biol. Res. 50:e6432. doi: 10.1590/1414-431X20176432, PMID: 29069229 PMC5649871

[ref21] Cureton K. J. Sparling P. B. Evans B. W. Johnson S. M. Kong U. D. Purvis J. W. (1978). Effect of experimental alterations in excess weight on aerobic capacity and distance running performance. Med. Sci. Sports 10, 194–199, PMID: 723510

[ref22] de Souza C. F. Stopa L. R. S. Santos G. F. Takasumi L. C. N. Martins A. B. Garnica-Siqueira M. C.. (2019). Estradiol protects against ovariectomy-induced susceptibility to the anabolic effects of glucocorticoids in rats. Life Sci. 218, 185–196. doi: 10.1016/j.lfs.2018.12.037, PMID: 30594666

[ref23] Del Coso J. Valero M. Lara B. Salinero J. J. Gallo-Salazar C. Areces F. (2016). Myosin light chain kinase (MLCK) gene influences exercise induced muscle damage during a competitive Marathon. PLoS One 11:e0160053. doi: 10.1371/journal.pone.0160053, PMID: 27483374 PMC4970719

[ref24] El Khoudary S. R. Ceponiene I. Samargandy S. Stein J. H. Li D. Tattersall M. C.. (2018). HDL (high-density lipoprotein) metrics and atherosclerotic risk in women. Arterioscler. Thromb. Vasc. Biol. 38, 2236–2244. doi: 10.1161/ATVBAHA.118.311017, PMID: 30026268 PMC6202150

[ref25] Erickson K. I. Voss M. W. Prakash R. S. Basak C. Szabo A. Chaddock L.. (2011). Exercise training increases size of hippocampus and improves memory. Proc. Natl. Acad. Sci. USA 108, 3017–3022. doi: 10.1073/pnas.1015950108, PMID: 21282661 PMC3041121

[ref26] Fernandez M. L. Murillo A. G. (2016). Postmenopausal women have higher HDL and decreased incidence of low HDL than premenopausal women with metabolic syndrome. Healthcare 4:20. doi: 10.3390/healthcare4010020, PMID: 27417608 PMC4934554

[ref27] Frasier C. R. Brown D. A. Sloan R. C. Hayes B. Stewart L. M. Patel H. D.. (2013). Stage of the estrous cycle does not influence myocardial ischemia-reperfusion injury in rats (*Rattus norvegicus*). Comp. Med. 63, 416–421, PMID: 24210018 PMC3796752

[ref28] Giles E. D. Jackman M. R. Johnson G. C. Schedin P. J. Houser J. L. MacLean P. S. (2010). Effect of the estrous cycle and surgical ovariectomy on energy balance, fuel utilization, and physical activity in lean and obese female rats. Am. J. Physiol. Regul. Integr. Comp. Physiol. 299, R1634–R1642. doi: 10.1152/ajpregu.00219.2010, PMID: 20926768 PMC3007190

[ref29] Gordon D. Scruton A. Barnes R. Baker J. Prado L. Merzbach V. (2018). The effects of menstrual cycle phase on the incidence of plateau at V˙O2max and associated cardiorespiratory dynamics. Clin. Physiol. Funct. Imaging 38, 689–698. doi: 10.1111/cpf.12469, PMID: 28906053

[ref30] Greendale G. A. Sternfeld B. Huang M. Han W. Karvonen-Gutierrez C. Ruppert K.. (2019). Changes in body composition and weight during the menopause transition. JCI Insight 4:e124865. doi: 10.1172/jci.insight.124865, PMID: 30843880 PMC6483504

[ref31] Heikkinen T. Puoliväli J. Liu L. Rissanen A. Tanila H. (2002). Effects of ovariectomy and estrogen treatment on learning and hippocampal neurotransmitters in mice. Horm. Behav. 41, 22–32. doi: 10.1006/hbeh.2001.1738, PMID: 11863380

[ref32] Hertrampf T. Degen G. H. Kaid A. A. Laudenbach-Leschowsky U. Seibel J. Di Virgilio A. L.. (2006). Combined effects of physical activity, dietary isoflavones and 17beta-estradiol on movement drive, body weight and bone mineral density in ovariectomized female rats. Planta Med. 72, 484–487. doi: 10.1055/s-2006-931579, PMID: 16773530

[ref33] Heymsfield S. B. Smith B. Dahle J. Kennedy S. Fearnbach N. Thomas D. M.. (2021). Resting energy expenditure: from cellular to whole-body level, a mechanistic historical perspective. Obesity 29, 500–511. doi: 10.1002/oby.23090, PMID: 33624441

[ref34] Heymsfield S. B. Thomas D. M. Bosy-Westphal A. Müller M. J. (2019). The anatomy of resting energy expenditure: body composition mechanisms. Eur. J. Clin. Nutr. 73, 166–171. doi: 10.1038/s41430-018-0319-3, PMID: 30254244 PMC6410366

[ref35] Ikemoto S. Panksepp J. (1999). The role of nucleus accumbens dopamine in motivated behavior: a unifying interpretation with special reference to reward-seeking. Brain Res. Brain Res. Rev. 31, 6–41. doi: 10.1016/s0165-0173(99)00023-5, PMID: 10611493

[ref36] Izumo N. Ishibashi Y. Ohba M. Morikawa T. Manabe T. (2012). Decreased voluntary activity and amygdala levels of serotonin and dopamine in ovariectomized rats. Behav. Brain Res. 227, 1–6. doi: 10.1016/j.bbr.2011.10.031, PMID: 22056749

[ref37] Jacobs B. L. Fornal C. A. (1997). Serotonin and motor activity. Curr. Opin. Neurobiol. 7, 820–825. doi: 10.1016/s0959-4388(97)80141-9 9464975

[ref38] Jarrard L. E. (1993). On the role of the hippocampus in learning and memory in the rat. Behav. Neural Biol. 60, 9–26. doi: 10.1016/0163-1047(93)90664-4 8216164

[ref39] Juppi H.-K. Sipilä S. Fachada V. Hyvärinen M. Cronin N. Aukee P.. (2022). Total and regional body adiposity increases during menopause-evidence from a follow-up study. Aging Cell 21:e13621. doi: 10.1111/acel.13621, PMID: 35509177 PMC9197413

[ref40] Kadi F. Karlsson C. Larsson B. Eriksson J. Larval M. Billig H.. (2002). The effects of physical activity and estrogen treatment on rat fast and slow skeletal muscles following ovariectomy. J. Muscle Res. Cell Motil. 23, 335–339. doi: 10.1023/a:1022071114344, PMID: 12630708

[ref41] Karppinen J. E. Wiklund P. Ihalainen J. K. Juppi H.-K. Isola V. Hyvärinen M.. (2023). Age but not menopausal status is linked to lower resting energy expenditure. J. Clin. Endocrinol. Metab. 108, 2789–2797. doi: 10.1210/clinem/dgad321, PMID: 37265230 PMC10584005

[ref42] Karvinen S. Jergenson M. J. Hyvärinen M. Aukee P. Tammelin T. Sipilä S.. (2019). Menopausal status and physical activity are independently associated with cardiovascular risk factors of healthy middle-aged women: cross-sectional and longitudinal evidence. Front. Endocrinol. 10:589. doi: 10.3389/fendo.2019.00589, PMID: 31543865 PMC6729112

[ref43] Karvinen S. Juppi H.-K. Le G. Cabelka C. A. Mader T. L. Lowe D. A.. (2021). Estradiol deficiency and skeletal muscle apoptosis: possible contribution of microRNAs. Exp. Gerontol. 147:111267. doi: 10.1016/j.exger.2021.111267, PMID: 33548486 PMC9897888

[ref44] Karvinen S. Silvennoinen M. Vainio P. Sistonen L. Koch L. G. Britton S. L.. (2016). Effects of intrinsic aerobic capacity, aging and voluntary running on skeletal muscle sirtuins and heat shock proteins. Exp. Gerontol. 79, 46–54. doi: 10.1016/j.exger.2016.03.015, PMID: 27038700

[ref45] Keuper M. Jastroch M. (2021). The good and the BAT of metabolic sex differences in thermogenic human adipose tissue. Mol. Cell. Endocrinol. 533:111337. doi: 10.1016/j.mce.2021.111337, PMID: 34062167

[ref46] Klass M. Duchateau J. Rabec S. Meeusen R. Roelands B. (2016). Noradrenaline reuptake inhibition impairs cortical output and limits endurance time. Med. Sci. Sports Exerc. 48, 1014–1023. doi: 10.1249/MSS.0000000000000879, PMID: 26784275

[ref47] Koch L. G. Britton S. L. (2001). Artificial selection for intrinsic aerobic endurance running capacity in rats. Physiol. Genomics 5, 45–52. doi: 10.1152/physiolgenomics.2001.5.1.45, PMID: 11161005

[ref48] Laakkonen E. K. Kulmala J. Aukee P. Hakonen H. Kujala U. M. Lowe D. A.. (2017). Female reproductive factors are associated with objectively measured physical activity in middle-aged women. PLoS One 12:e0172054. doi: 10.1371/journal.pone.0172054, PMID: 28225786 PMC5321412

[ref49] Lai S. Collins B. C. Colson B. A. Kararigas G. Lowe D. A. (2016). Estradiol modulates myosin regulatory light chain phosphorylation and contractility in skeletal muscle of female mice. Am. J. Physiol. Endocrinol. Metab. 310, E724–E733. doi: 10.1152/ajpendo.00439.2015, PMID: 26956186 PMC4867308

[ref50] Lambrinoudaki I. Paschou S. A. Armeni E. Goulis D. G. (2022). The interplay between diabetes mellitus and menopause: clinical implications. Nat. Rev. Endocrinol. 18, 608–622. doi: 10.1038/s41574-022-00708-0, PMID: 35798847

[ref51] Lee H. R. Shin J. Han K. Chang J. Jeong S.-M. Chon S. J.. (2021). Obesity and risk of diabetes mellitus by menopausal status: a Nationwide cohort study. J. Clin. Med. 10:5189. doi: 10.3390/jcm10215189, PMID: 34768709 PMC8584465

[ref52] Lensu S. Pariyani R. Mäkinen E. Yang B. Saleem W. Munukka E.. (2020). Prebiotic Xylo-oligosaccharides ameliorate high-fat-diet-induced hepatic steatosis in rats. Nutrients 12:3225. doi: 10.3390/nu12113225, PMID: 33105554 PMC7690286

[ref53] Ley C. J. Lees B. Stevenson J. C. (1992). Sex- and menopause-associated changes in body-fat distribution. Am. J. Clin. Nutr. 55, 950–954. doi: 10.1093/ajcn/55.5.950, PMID: 1570802

[ref54] Livingstone C. Collison M. (2002). Sex steroids and insulin resistance. Clin. Sci. (Lond.) 102, 151–166. doi: 10.1042/cs1020151 11834135

[ref55] Long T. Yao J. K. Li J. Kirshner Z. Z. Nelson D. Dougherty G. G.. (2018). Comparison of transitional vs surgical menopause on monoamine and amino acid levels in the rat brain. Mol. Cell. Endocrinol. 476, 139–147. doi: 10.1016/j.mce.2018.05.003, PMID: 29738870 PMC6120792

[ref56] Lovejoy J. C. Champagne C. M. de Jonge L. Xie H. Smith S. R. (2008). Increased visceral fat and decreased energy expenditure during the menopausal transition. Int. J. Obes. 32, 949–958. doi: 10.1038/ijo.2008.25, PMID: 18332882 PMC2748330

[ref57] Mandrup C. M. Egelund J. Nyberg M. Lundberg Slingsby M. H. Andersen C. B. Løgstrup S.. (2017). Effects of high-intensity training on cardiovascular risk factors in premenopausal and postmenopausal women. Am. J. Obstet. Gynecol. 216, 384.e1–384.e11. doi: 10.1016/j.ajog.2016.12.017, PMID: 28024987

[ref58] McElroy J. F. Wade G. N. (1987). Short- and long-term effects of ovariectomy on food intake, body weight, carcass composition, and brown adipose tissue in rats. Physiol. Behav. 39, 361–365. doi: 10.1016/0031-9384(87)90235-6, PMID: 3575476

[ref59] McLean A. C. Valenzuela N. Fai S. Bennett S. A. L. (2012). Performing vaginal lavage, crystal violet staining, and vaginal cytological evaluation for mouse estrous cycle staging identification. J. Vis. Exp. 67:e4389. doi: 10.3791/4389, PMID: 23007862 PMC3490233

[ref60] Meeusen R. De Meirleir K. (1995). Exercise and brain neurotransmission. Sports Med. 20, 160–188. doi: 10.2165/00007256-199520030-00004 8571000

[ref61] Meyniel F. Goodwin G. M. Deakin J. W. Klinge C. MacFadyen C. Milligan H.. (2016). A specific role for serotonin in overcoming effort cost. eLife 5:e17282. doi: 10.7554/eLife.17282, PMID: 27824554 PMC5100997

[ref62] Min W. Fang P. Huang G. Shi M. Zhang Z. (2018). The decline of whole-body glucose metabolism in ovariectomized rats. Exp. Gerontol. 113, 106–112. doi: 10.1016/j.exger.2018.09.027, PMID: 30292771

[ref63] Moncrieff J. Cooper R. E. Stockmann T. Amendola S. Hengartner M. P. Horowitz M. A. (2022). The serotonin theory of depression: a systematic umbrella review of the evidence. Mol. Psychiatry 28, 3243–3256. doi: 10.1038/s41380-022-01661-0, PMID: 35854107 PMC10618090

[ref64] Moran A. L. Warren G. L. Lowe D. A. (2006). Removal of ovarian hormones from mature mice detrimentally affects muscle contractile function and myosin structural distribution. J. Appl. Physiol. 100, 548–559. doi: 10.1152/japplphysiol.01029.2005, PMID: 16254070

[ref65] Moschinger M. Hilse K. E. Rupprecht A. Zeitz U. Erben R. G. Rülicke T.. (2019). Age-related sex differences in the expression of important disease-linked mitochondrial proteins in mice. Biol. Sex Differ. 10:56. doi: 10.1186/s13293-019-0267-1, PMID: 31806023 PMC6896328

[ref66] Müller T. D. Klingenspor M. Tschöp M. H. (2021). Publisher correction: revisiting energy expenditure: how to correct mouse metabolic rate for body mass. Nat. Metab. 3:1433. doi: 10.1038/s42255-021-00485-6, PMID: 34625742

[ref67] Owesson-White C. A. Cheer J. F. Beyene M. Carelli R. M. Wightman R. M. (2008). Dynamic changes in accumbens dopamine correlate with learning during intracranial self-stimulation. Proc. Natl. Acad. Sci. USA 105, 11957–11962. doi: 10.1073/pnas.0803896105, PMID: 18689678 PMC2575325

[ref68] Pansini F. Bonaccorsi G. Calisesi M. Campobasso C. Franze G. P. Gilli G.. (1993). Influence of spontaneous and surgical menopause on atherogenic metabolic risk. Maturitas 17, 181–190. doi: 10.1016/0378-5122(93)90045-j, PMID: 8133792

[ref69] Park Y.-M. Kanaley J. A. Padilla J. Zidon T. Welly R. J. Will M. J.. (2016). Effects of intrinsic aerobic capacity and ovariectomy on voluntary wheel running and nucleus accumbens dopamine receptor gene expression. Physiol. Behav. 164, 383–389. doi: 10.1016/j.physbeh.2016.06.006, PMID: 27297873 PMC4983202

[ref70] Peacock K. Ketvertis K. M. (2023). Menopause.

[ref71] Pedersen S. B. Bruun J. M. Kristensen K. Richelsen B. (2001). Regulation of UCP1, UCP2, and UCP3 mRNA expression in brown adipose tissue, white adipose tissue, and skeletal muscle in rats by estrogen. Biochem. Biophys. Res. Commun. 288, 191–197. doi: 10.1006/bbrc.2001.5763, PMID: 11594772

[ref72] Péronnet F. Haman F. (2019). Low capacity to oxidize fat and body weight. Obes. Rev. 20, 1367–1383. doi: 10.1111/obr.12910 31353786

[ref73] Pettee Gabriel K. Mason J. M. Sternfeld B. (2015). Recent evidence exploring the associations between physical activity and menopausal symptoms in midlife women: perceived risks and possible health benefits. Womens Midlife Health 1:1. doi: 10.1186/s40695-015-0004-9, PMID: 30766688 PMC6214216

[ref74] Phung L. A. Karvinen S. M. Colson B. A. Thomas D. D. Lowe D. A. (2018). Age affects myosin relaxation states in skeletal muscle fibers of female but not male mice. PLoS One 13:e0199062. doi: 10.1371/journal.pone.0199062, PMID: 30226869 PMC6143227

[ref75] Pontzer H. Yamada Y. Sagayama H. Ainslie P. N. Andersen L. F. Anderson L. J.. (2021). Daily energy expenditure through the human life course. Science 373, 808–812. doi: 10.1126/science.abe5017, PMID: 34385400 PMC8370708

[ref76] Qaisar R. Renaud G. Hedstrom Y. Pöllänen E. Ronkainen P. Kaprio J.. (2013). Hormone replacement therapy improves contractile function and myonuclear organization of single muscle fibres from postmenopausal monozygotic female twin pairs. J. Physiol. 591, 2333–2344. doi: 10.1113/jphysiol.2012.250092, PMID: 23459759 PMC3650698

[ref77] Queathem E. D. Welly R. J. Clart L. M. Rowles C. C. Timmons H. Fitzgerald M.. (2021). White adipose tissue depots respond to chronic Beta-3 adrenergic receptor activation in a sexually dimorphic and depot divergent manner. Cells 10:3453. doi: 10.3390/cells10123453, PMID: 34943961 PMC8700379

[ref78] Rayment I. Holden H. M. Whittaker M. Yohn C. B. Lorenz M. Holmes K. C.. (1993). Structure of the actin-myosin complex and its implications for muscle contraction. Science 261, 58–65. doi: 10.1126/science.8316858, PMID: 8316858

[ref79] Riley C. L. Dao C. Kenaston M. A. Muto L. Kohno S. Nowinski S. M.. (2016). The complementary and divergent roles of uncoupling proteins 1 and 3 in thermoregulation. J. Physiol. 594, 7455–7464. doi: 10.1113/JP272971, PMID: 27647490 PMC5157057

[ref80] Rodier W. I. (1971). Progesterone-estrogen interactions in the control of activity-wheel running in the female rat. J. Comp. Physiol. Psychol. 74, 365–373. doi: 10.1037/h0030568, PMID: 5546884

[ref81] Rogers N. H. Perfield J. W. Strissel K. J. Obin M. S. Greenberg A. S. (2009). Reduced energy expenditure and increased inflammation are early events in the development of ovariectomy-induced obesity. Endocrinology 150, 2161–2168. doi: 10.1210/en.2008-1405, PMID: 19179442 PMC2671894

[ref82] Rousset S. Alves-Guerra M.-C. Mozo J. Miroux B. Cassard-Doulcier A.-M. Bouillaud F.. (2004). The biology of mitochondrial uncoupling proteins. Diabetes 53, S130–S135. doi: 10.2337/diabetes.53.2007.s130 14749278

[ref83] Rybaczyk L. A. Bashaw M. J. Pathak D. R. Moody S. M. Gilders R. M. Holzschu D. L. (2005). An overlooked connection: serotonergic mediation of estrogen-related physiology and pathology. BMC Womens Health 5:12. doi: 10.1186/1472-6874-5-12, PMID: 16368009 PMC1327664

[ref84] Shimizu H. Shimomura Y. Takahashi M. Uehara Y. Fukatsu A. Sato N. (1991). Altered ambulatory activity and related brain monoamine metabolism in genetically obese Zucker rats. Exp. Clin. Endocrinol. 97, 39–44. doi: 10.1055/s-0029-1211036, PMID: 1864312

[ref85] Silvennoinen M. Rantalainen T. Kainulainen H. (2014). Validation of a method to measure total spontaneous physical activity of sedentary and voluntary running mice. J. Neurosci. Methods 235, 51–58. doi: 10.1016/j.jneumeth.2014.06.027, PMID: 24979727

[ref86] Soares D. D. Lima N. R. V. Coimbra C. C. Marubayashi U. (2003). Evidence that tryptophan reduces mechanical efficiency and running performance in rats. Pharmacol. Biochem. Behav. 74, 357–362. doi: 10.1016/s0091-3057(02)01003-1, PMID: 12479955

[ref87] Sotocinal S. G. Sorge R. E. Zaloum A. Tuttle A. H. Martin L. J. Wieskopf J. S.. (2011). The rat grimace scale: a partially automated method for quantifying pain in the laboratory rat via facial expressions. Mol. Pain 7:55. doi: 10.1186/1744-8069-7-55, PMID: 21801409 PMC3163602

[ref88] Švob Štrac D. Pivac N. Mück-Šeler D. (2016). The serotonergic system and cognitive function. Transl. Neurosci. 7, 35–49. doi: 10.1515/tnsci-2016-0007, PMID: 28123820 PMC5017596

[ref89] Tawfik S. H. Mahmoud B. F. Saad M. I. Shehata M. Kamel M. A. Helmy M. H. (2015). Similar and additive effects of ovariectomy and diabetes on insulin resistance and lipid metabolism. Biochem. Res. Int. 2015:567945. doi: 10.1155/2015/567945, PMID: 25834745 PMC4365318

[ref90] Tsai S.-W. Chen C.-J. Chen H.-L. Chen C.-M. Chang Y.-Y. (2012). Effects of treadmill running on rat gastrocnemius function following botulinum toxin a injection. J. Orthop. Res. 30, 319–324. doi: 10.1002/jor.21509, PMID: 21815203

[ref91] van Galen K. A. Ter Horst K. W. Serlie M. J. (2021). Serotonin, food intake, and obesity. Obes. Rev. 22:e13210. doi: 10.1111/obr.13210, PMID: 33559362 PMC8243944

[ref92] Wissink S. van der Burg B. Katzenellenbogen B. S. van der Saag P. T. (2001). Synergistic activation of the serotonin-1A receptor by nuclear factor-kappa B and estrogen. Mol. Endocrinol. 15, 543–552. doi: 10.1210/mend.15.4.0629, PMID: 11266506

[ref93] Woodard G. A. Brooks M. M. Barinas-Mitchell E. Mackey R. H. Matthews K. A. Sutton-Tyrrell K. (2011). Lipids, menopause, and early atherosclerosis in study of Women’s health across the nation heart women. Menopause 18, 376–384. doi: 10.1097/gme.0b013e3181f6480e, PMID: 21107300 PMC3123389

[ref94] Xu Y. López M. (2018). Central regulation of energy metabolism by estrogens. Mol. Metab. 15, 104–115. doi: 10.1016/j.molmet.2018.05.012, PMID: 29886181 PMC6066788

[ref95] Young S. N. (2007). How to increase serotonin in the human brain without drugs. J. Psychiatry Neurosci. 32, 394–399, PMID: 18043762 PMC2077351

[ref96] Yousefzadeh N. Kashfi K. Jeddi S. Ghasemi A. (2020). Ovariectomized rat model of osteoporosis: a practical guide. EXCLI J. 19, 89–107. doi: 10.17179/excli2019-1990, PMID: 32038119 PMC7003643

