# Supplementary material for: Estrogen deficiency reduces maximal running capacity and affects serotonin levels differently in the hippocampus and nucleus accumbens in response to acute exercise
Source: Front Neurosci. 2024 Jun 25;18:1399229. doi: 10.3389/fnins.2024.1399229 (PMC11231437; doi:10.3389/fnins.2024.1399229)
Supplement: Supplementary file 1 [file Data_Sheet_1.pdf]

Supplementary Material

1 Supplementary Figures

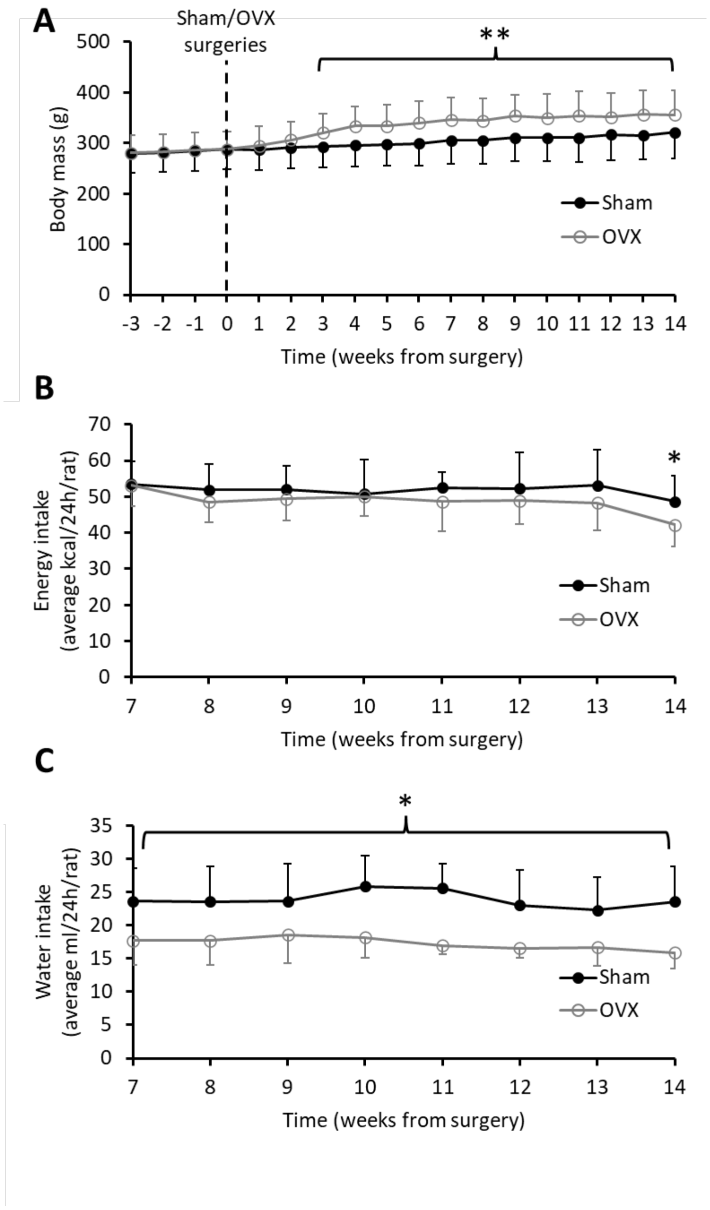

Supplementary Figure 1. Body mass (**A**) measured once a week 3 weeks before and 14 after the surgeries. Energy intake (**B**), and water intake (**C**) measured once a week from pair-housed rats between weeks 7-14 after the surgery. Figures show means with SD. *p≤0.050, **p≤0.010.

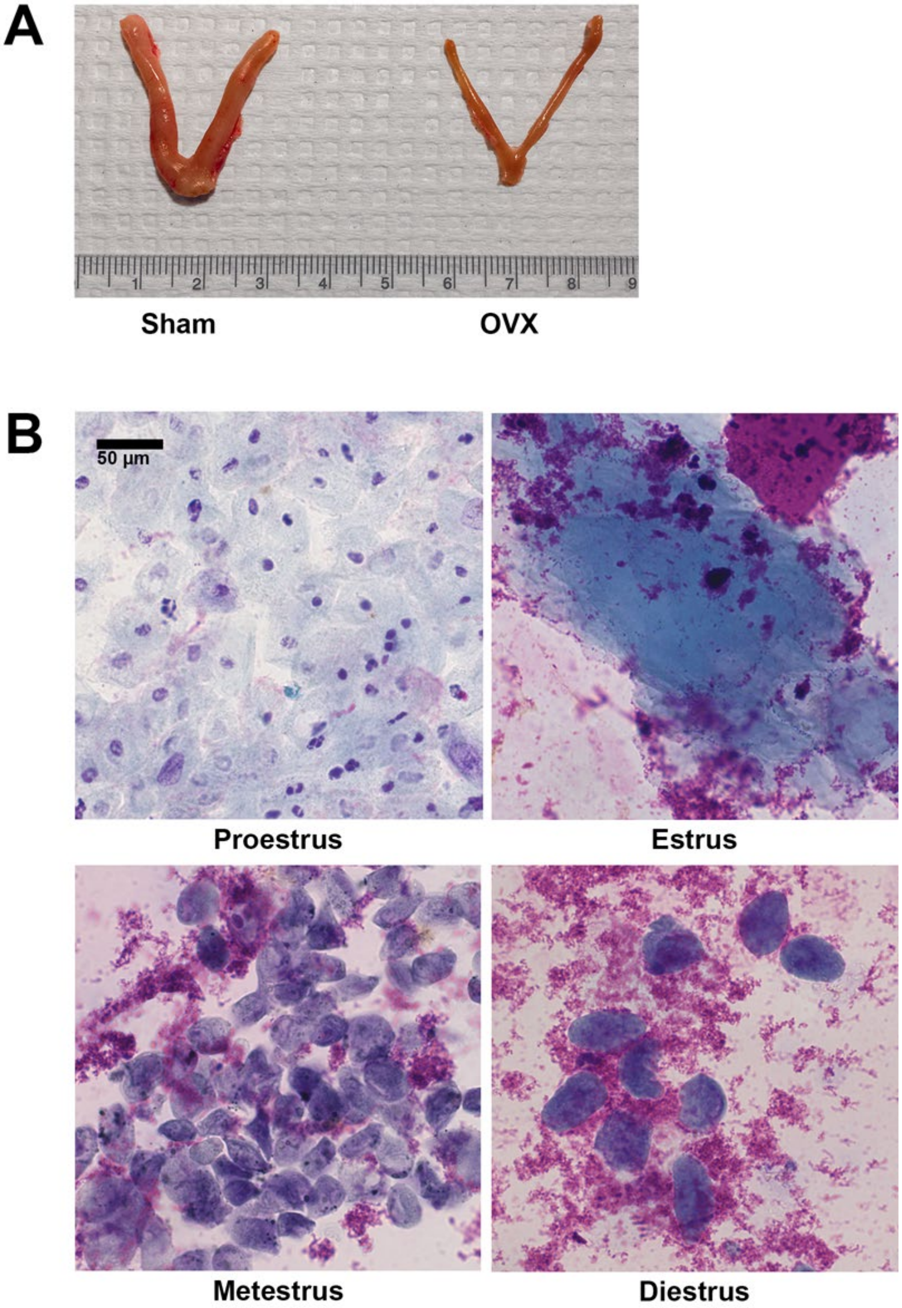

Supplementary Figure 2. Representative images of uteri (A) and vaginal cytology samples of the four estrous cycle phases (B). Representative images show uterine involution after OVX compared with sham surgery (A). Proestrus was determined based on the presence of nucleated epithelial cells and estrus based on anucleated, cornified epithelial cells. Metestrus was determined by the presence of anucleated epithelial cells with neutrophils and diestrus with predominance of leucocytes accompanied with both nucleated and anucleated epithelial cells.

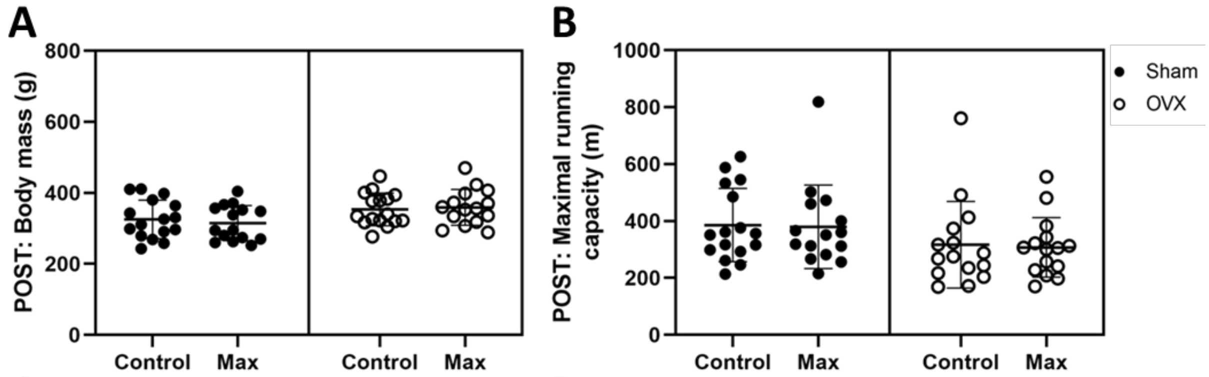

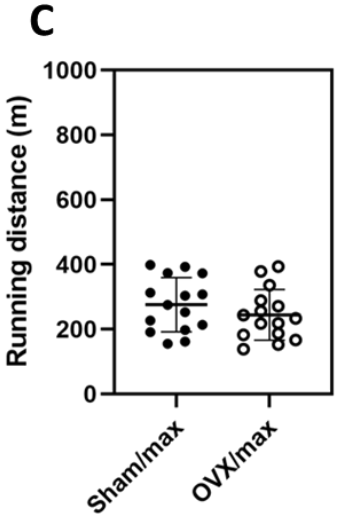

D

Main effects	OVX
POST Body mass (g)	**0.006**
POST Max running capacity (m)	**0.013**
END Running distance (m)	0.299

Supplementary Figure 3. Body mass (A), maximal running capacity (B), and running distance (C) right before euthanasia of the max groups. Main effect of OVX on the measured parameters (D). Figures show individual samples with mean and SD.

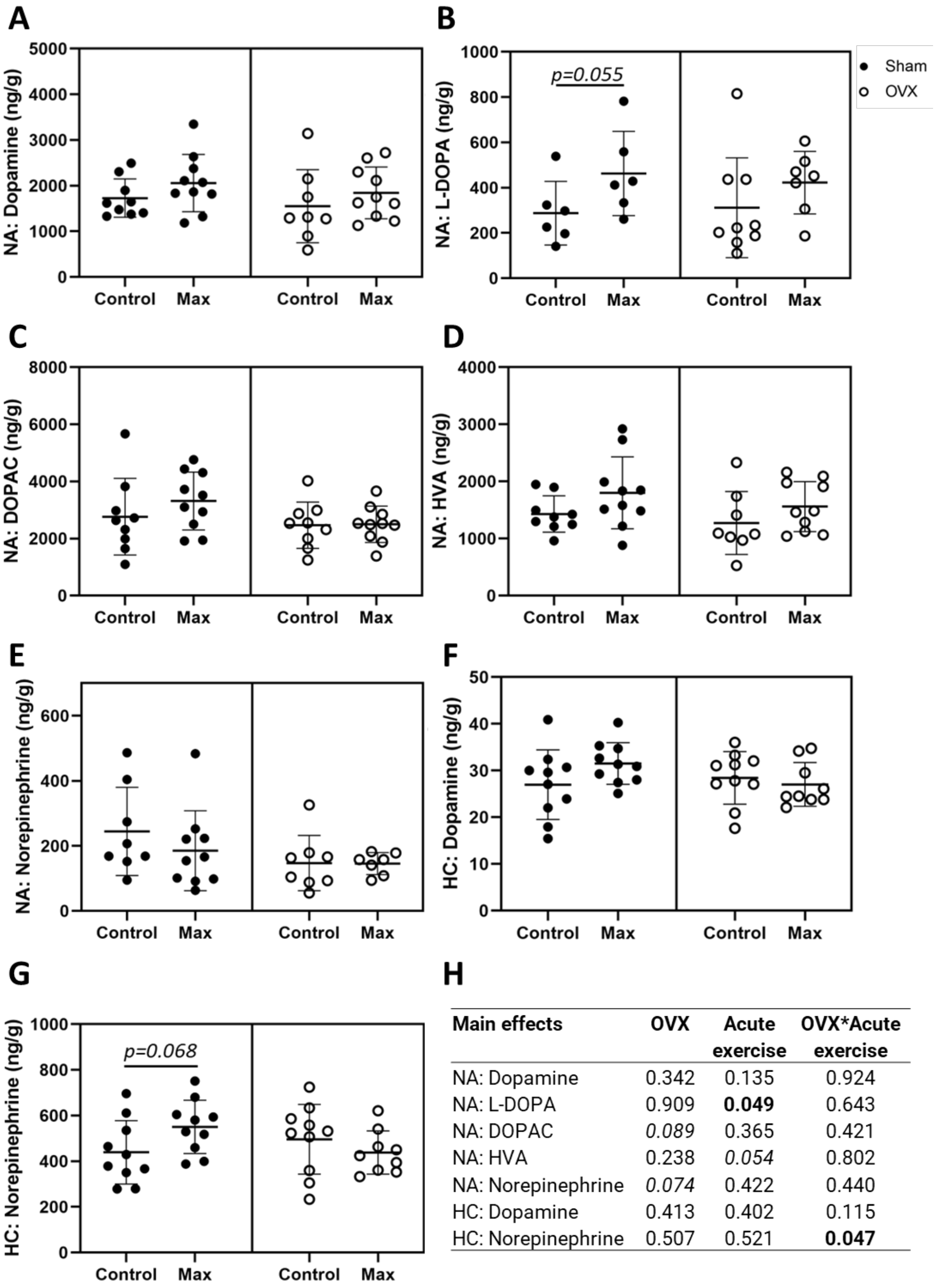

Main effects	OVX	Acute exercise	OVX*Acute exercise
NA: Dopamine	0.342	0.135	0.924
NA: L-DOPA	0.909	**0.049**	0.643
NA: DOPAC	*0.089*	0.365	0.421
NA: HVA	0.238	*0.054*	0.802
NA: Norepinephrine	*0.074*	0.422	0.440
HC: Dopamine	0.413	0.402	0.115
HC: Norepinephrine	0.507	0.521	**0.047**

Supplementary Figure 4. Dopamine (A), L-DOPA (B), DOPAC (C), homovalinnic acid (HVA) (D), and norepinephrine (E) levels in brain nucleus accumbens (NA) and dopamine (F) and norepinephrine (G) levels in hippocampus (HC) in sham and OVX rats. The main effects of OVX, acute exercise and their interaction on the measured neurochemical markers (H). Figures show individual samples with mean and SD.

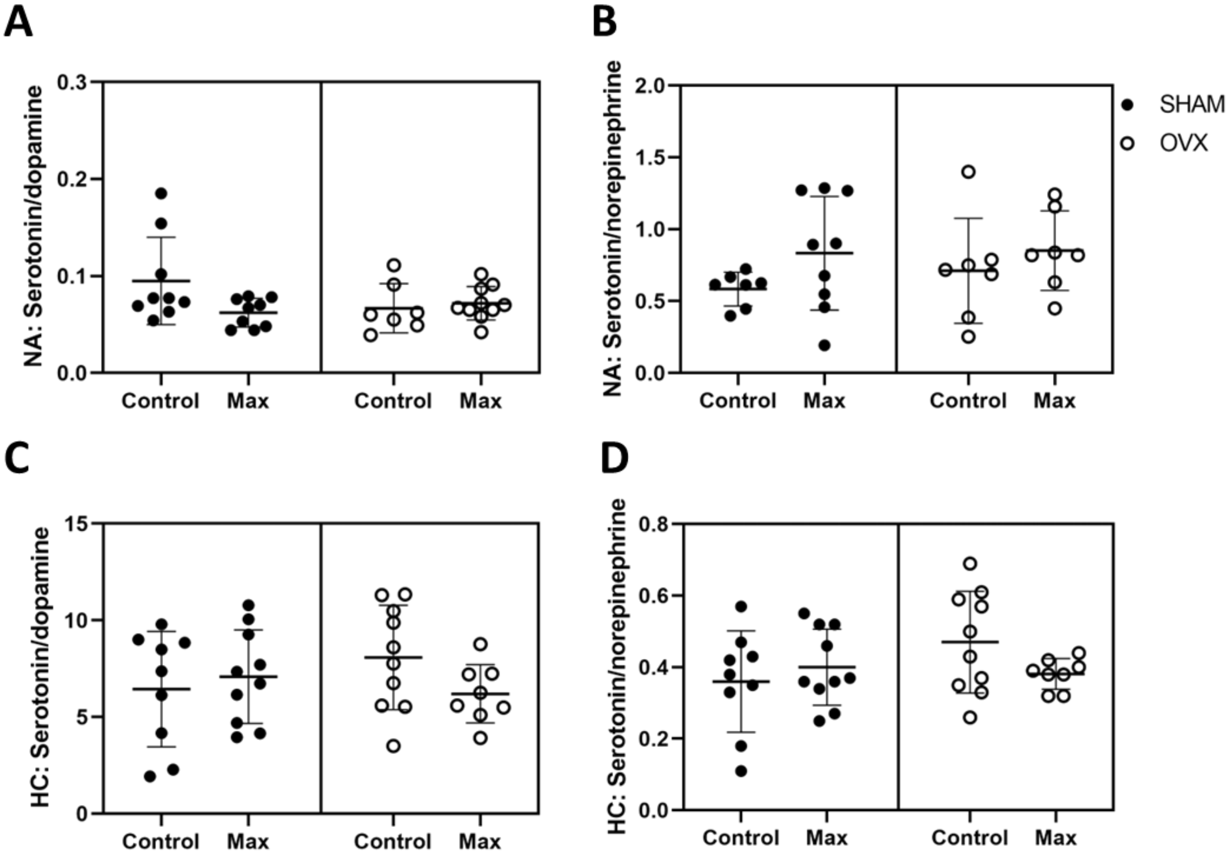

E

Main effects	OVX	Acute exercise	OVX*Acute exercise
NA: Serotonin/dopamine	0.350	0.150	*0.056*
NA: Serotonin/norepinephrine	0.534	0.107	0.640
HC: Serotonin/dopamine	0.656	0.457	0.136
HC: Serotonin/norepinephrine	0.252	0.552	0.107

Supplementary Figure 5. Serotonin/dopamine (A,C) and serotonin/ norepinephrine (B,D) ratios in brain nucleus accumbens (NA) and hippocampus (HC). The main effects of OVX, acute exercise and their interaction on the measured ratios (E). Figures show individual samples with mean and SD.

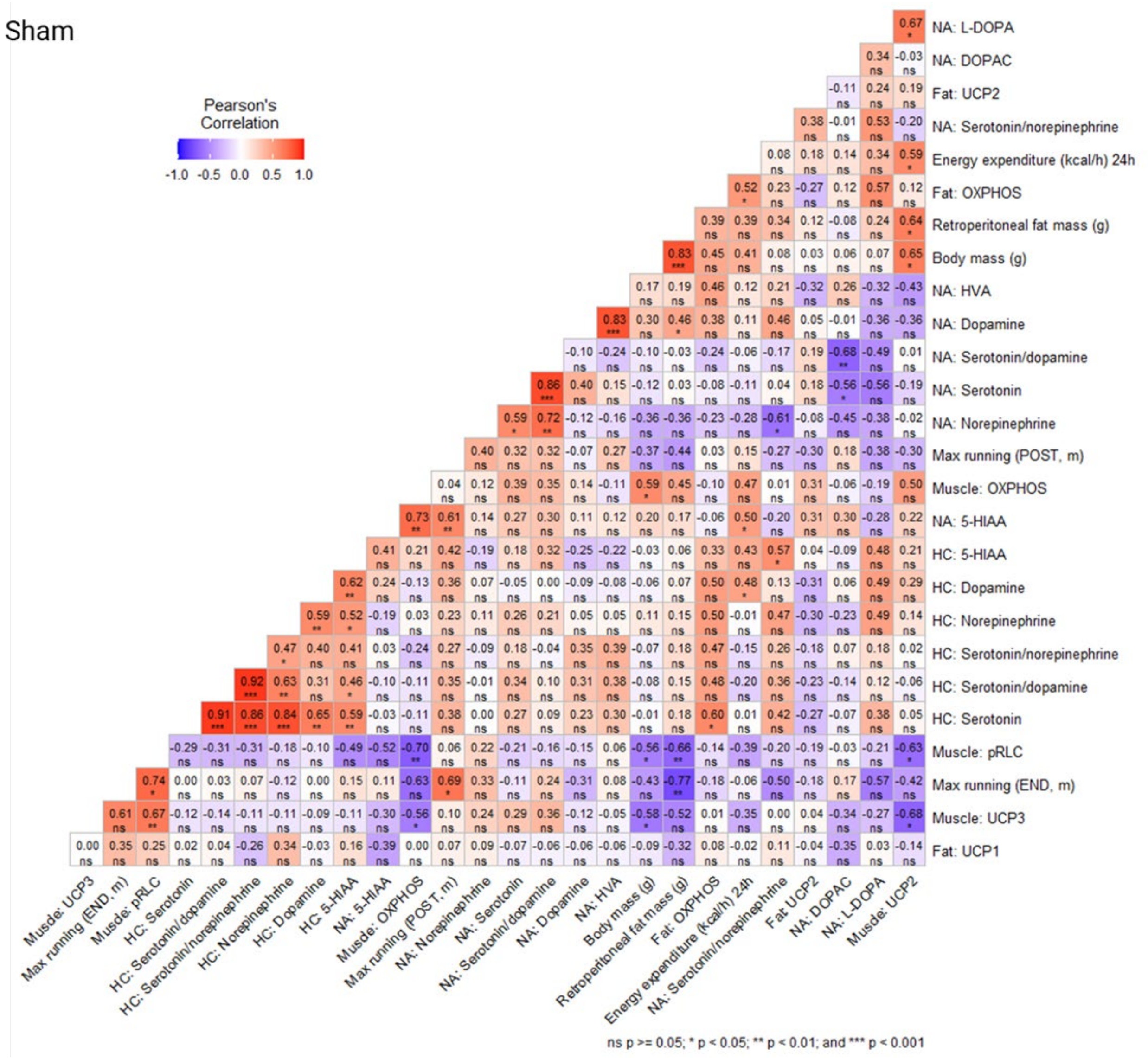

Supplementary Figure 6. Correlation coefficients (Pearson's correlation) in the sham group of brain neurochemical markers in the nucleus accumbens (NA) and hippocampus (HC), protein levels in muscle (gastrocnemius), adipose tissue (retroperitoneal deposit), body mass, retroperitoneal fat mass, maximal running capacity (POST, END), and 24h energy expenditure (kcal/h).

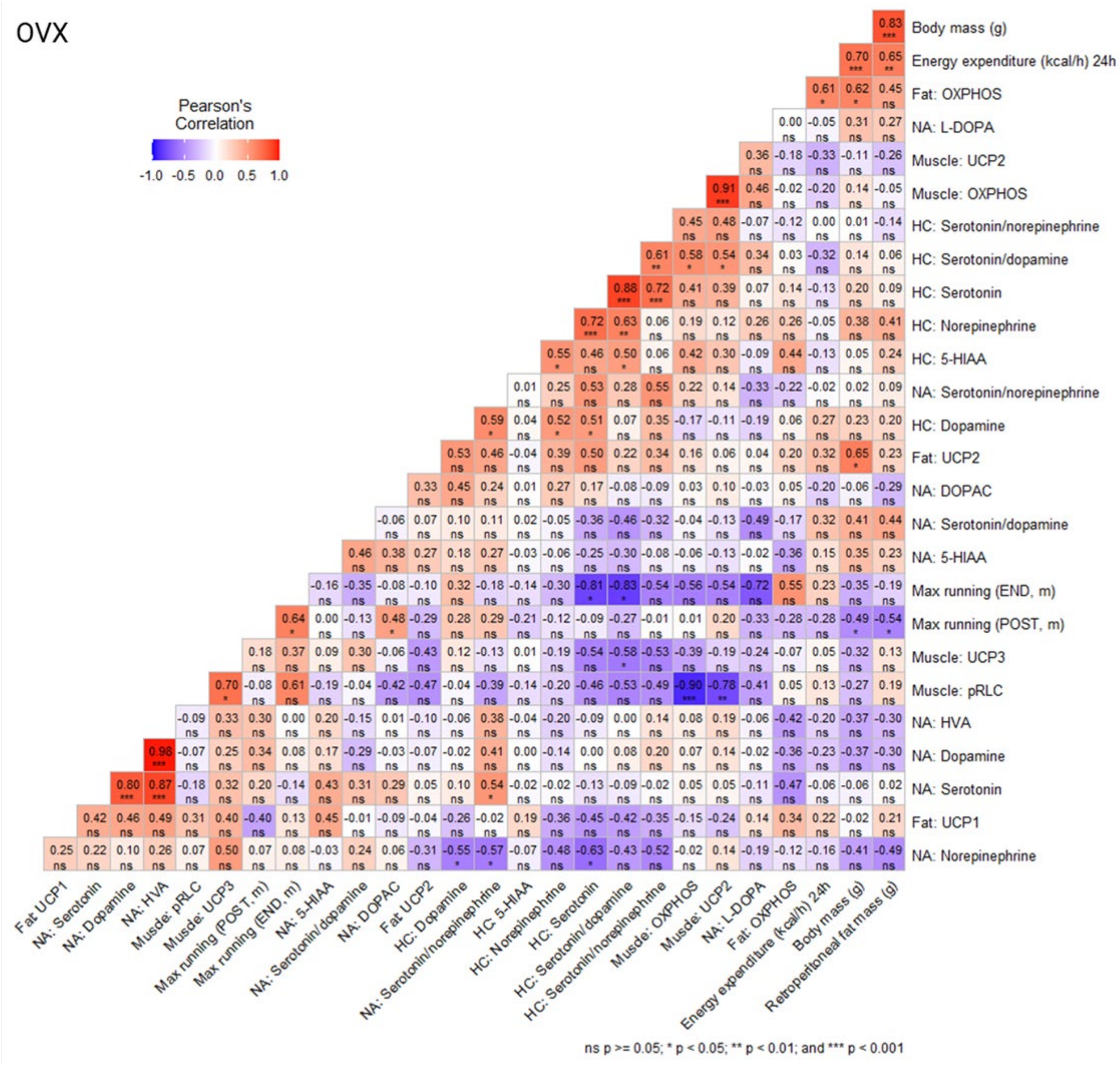

Supplementary Figure 7. Correlation coefficients (Pearson's correlation) in the OVX group of brain neurochemical markers in the nucleus accumbens (NA) and hippocampus (HC), protein levels in muscle (gastrocnemius), adipose tissue (retroperitoneal deposit), body mass, retroperitoneal fat mass, maximal running capacity (POST, END), and 24h energy expenditure (kcal/h).

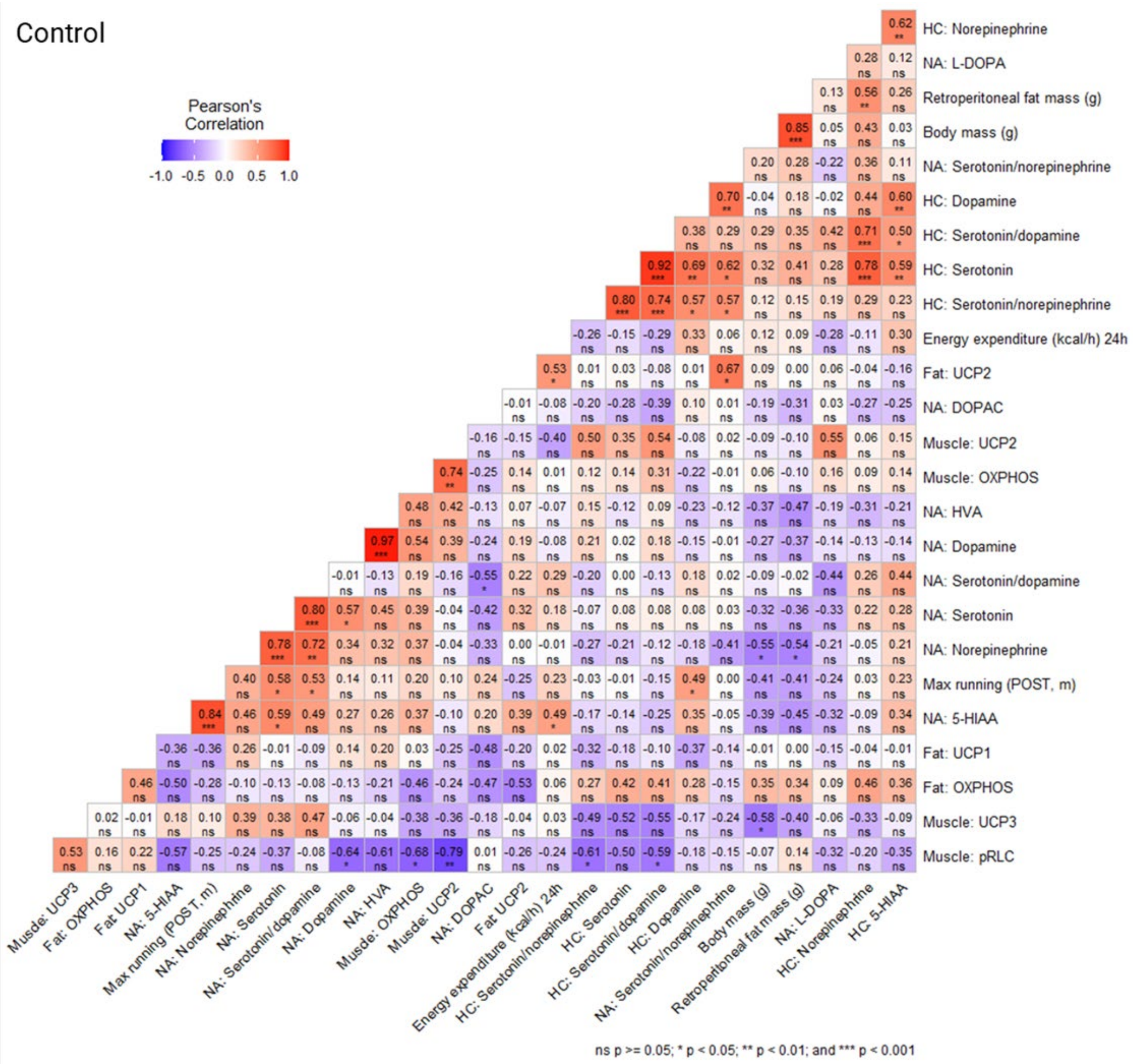

Supplementary Figure 8. Correlation coefficients (Pearson's correlation) in the control group (no acute exercise stimulus) of brain neurochemical markers in the nucleus accumbens (NA) and hippocampus (HC), protein levels in muscle (gastrocnemius), adipose tissue (retroperitoneal deposit), body mass, retroperitoneal fat mass, maximal running capacity (POST, END), and 24h energy expenditure (kcal/h).

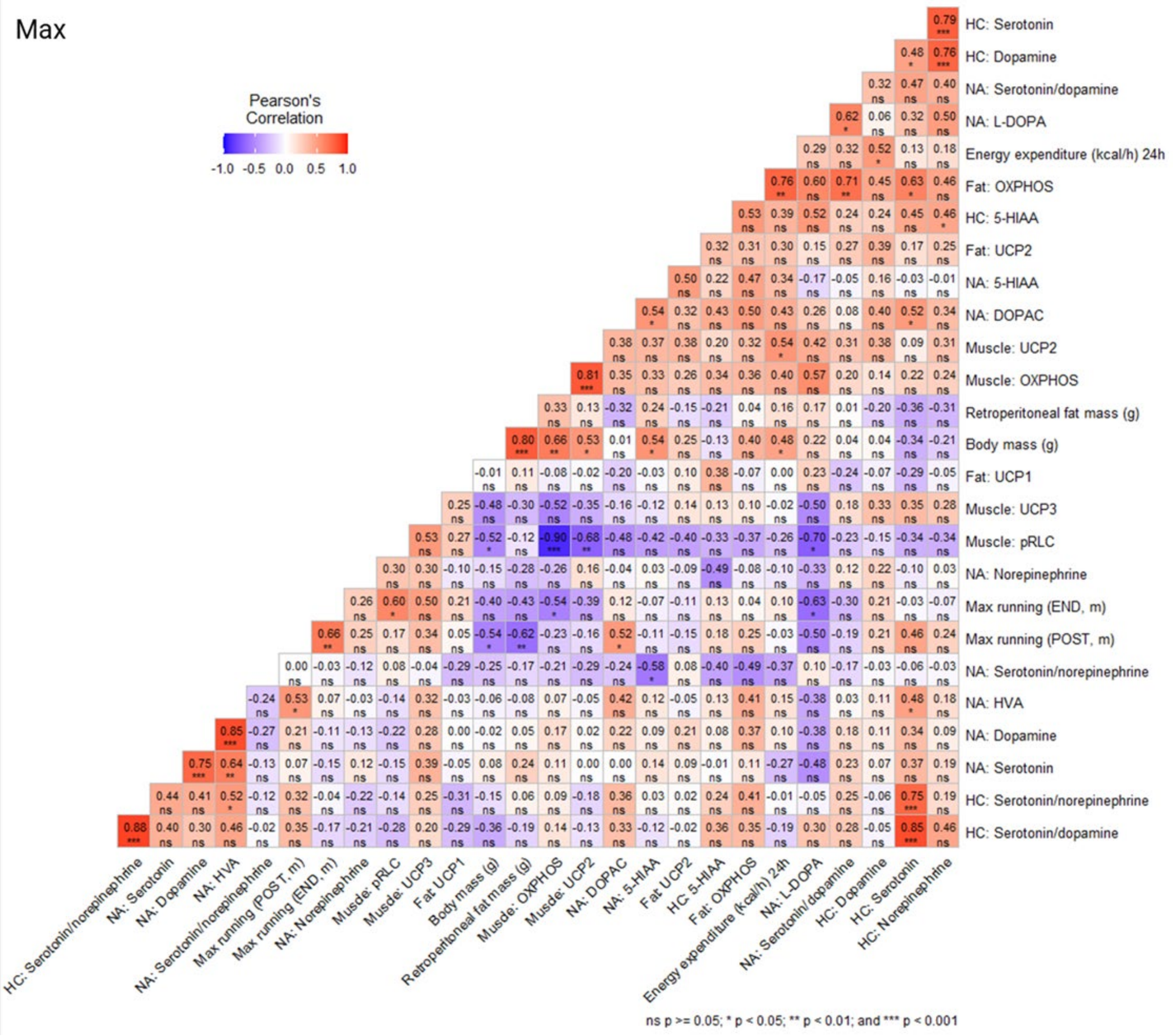

Supplementary Figure 9. Correlation coefficients (Pearson's correlation) in the max group (acute exercise stimulus) of brain neurochemical markers in the nucleus accumbens (NA) and hippocampus (HC), protein levels in muscle (gastrocnemius), adipose tissue (retroperitoneal deposit), body mass, retroperitoneal fat mass, maximal running capacity (POST, END), and 24h energy expenditure (kcal/h).
